# Development of Dimethylsulfonium Probes for Broad Profiling of Methyllysine Reader Proteins

**DOI:** 10.1002/advs.202517751

**Published:** 2025-11-29

**Authors:** Jinyu Yang, Yihang Xiao, Yingxiao Gao, Mingxuan Wu

**Affiliations:** ^1^ Department of Chemistry Zhejiang University Hangzhou Zhejiang 310027 China; ^2^ Key Laboratory of Precise Synthesis of Functional Molecules of Zhejiang Province Department of Chemistry School of Science Westlake University Hangzhou Zhejiang 310030 China; ^3^ Westlake Laboratory of Life Sciences and Biomedicine Hangzhou Zhejiang 310024 China; ^4^ Institute of Natural Sciences Westlake Institute for Advanced Study Hangzhou Zhejiang 310024 China

**Keywords:** crosslinking probe, lysine methylation, proteomic profiling, readers, sulfonium

## Abstract

Lysine methylation is a crucial post‐translational modification regulating various cellular processes. Reader proteins recognize specific methylated proteins as key mediators for the biological function of lysine methylation. Due to a correlation between reader activity and cell signaling of disorders, readers serve as attractive therapeutic targets. Despite proteomic advances identifying thousands of methylation sites, far fewer methyllysine readers have been characterized. Current research relies exclusively on site‐specific probes, which are restricted to individual sites but incapable of global profiling. Here, an oligoglycine‐based dimethylsulfonium peptide is reported as a general probe that is capable of binding and crosslinking to readers without bias toward particular sites or domains. The general reactivity to methyllysine readers in vitro is first demonstrated. The probe to cell nuclei is next applied, and the general probe enables identification of site‐specific readers by methyllysine peptide competition. In addition, the probe shows the potential for assessment of reader inhibitor activity and selectivity. Furthermore, bioinformatic analysis is performed to predict potential readers in the human genome, and global profiling of the potential readers in the nuclear proteome was achieved using the general probe. Thus, such a sulfonium probe provides a novel tool for global reader profiling, complementing the site‐specific probes.

## Graphical abstract

Development of oligoglycine‐based dimethylsulfonium probes for unbiased crosslinking to methyllysine readers. The general probe facilitates profiling of site‐specific methyllysine readers, evaluation of selectivity and activity of reader inhibitors, and global profiling of methyllysine readers. This strategy provides a versatile tool for the investigation of methyllysine readers.

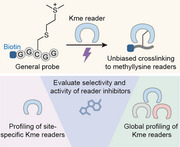

## 1 Introduction

Lysine methylation, as a common and important post‐translational modification (PTM), plays significant roles in gene transcription regulation, cell cycle control, DNA damage repair, and other cellular processes.^[^ ^1^, ^2^, ^3^, ^4^, ^5^, ^6^ ^]^ For instance, histone H3 lysine 4 trimethylation (H3K4me3) is involved in gene transcriptional activation, while H3K27me3 is related to gene transcriptional repression.^[^ ^1^ ^]^ Abnormalities in lysine methylation are also associated with the occurrence and development of diseases.^[^ ^7^, ^8^ ^]^ For example, aberrant hypermethylation at the H3K79 site can activate the transcription of genes such as MLL, thereby leading to the development of leukemia.^[^ ^9^, ^10^ ^]^

**Figure 1:**
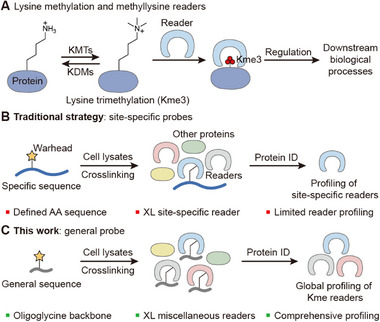
Introduction of methyllysine readers and strategies for reader investigation. A) Lysine methylation is dynamically regulated by lysine methyltransferases (KMTs) and lysine demethylases (KDMs). Readers recognize lysine methylation and regulate downstream biological processes. B) Schematic illustration of the identification of site‐specific readers by site‐specific probes. Protein ID: protein identification, AA: amino acid, XL: crosslink. C) In this work, the general probe allows global profiling of methyllysine readers.

Reader proteins, which specifically recognize methylation modifications at specific sites, are crucial for the biological functions of lysine methylation (**Figure** **1**A).^[^ ^11^, ^12^, ^13^, ^14^ ^]^ For example, BPTF is a reader of H3K4me3 that is in the NURF complex for RNA polymerase II activities.^[^ ^15^ ^]^ CBX1 is a reader of H3K9me3 that promotes liquid‐liquid phase separation for the formation of heterochromatin. As a result, the local genes in chromatin are condensed and not accessible to transcription.^[^ ^16^, ^17^, ^18^ ^]^ In addition, readers also play important roles in tumorigenesis. For example, EED, a subunit of polycomb repressive complex 2 (PRC2), recognizes H3K27me3 and thereby enhances the methyltransferase activity of EZH2, promoting further methylation of adjacent H3K27. As a result, DNA repair genes and tumor suppressor genes were silenced.^[^ ^19^ ^]^ Therefore, methylation readers are potential drug targets, and ligands and inhibitors of methyllysine readers have been continuously developed.^[^ ^20^, ^21^, ^22^, ^23^, ^24^, ^25^ ^]^ One example is EED226, which effectively inhibits PRC2 activity and suppresses tumor growth in mouse models.^[^ ^26^ ^]^ Building on the structure of EED226, researchers performed further structural optimization and developed MAK683 as a potential anticancer agent.^[^ ^27^ ^]^ Therefore, identification and investigation of readers will contribute to elucidating the functional roles and regulatory mechanisms of lysine methylation and provide theoretical support for the development of inhibitors and therapeutic strategies.

Although some histone methyllysine readers have been reported, there is still a substantial gap between the identification of methyllysine sites and the readers. Advances in methyllysine proteomics have revealed that such modification occurs across tens of thousands of sites in human cells, spanning not only histones but also a vast array of non‐histone proteins.^[^ ^28^ ^]^ It is highly demanding to discover many more readers to uncover the biology of lysine methylation, but the current methods cannot meet the requirement. A typical way to identify site‐specific readers is design and synthesis of probes with specific sequences, and the crosslinked readers are identified by proteomic approaches (Figure 1B).^[^ ^29^, ^30^, ^31^, ^32^, ^33^, ^34^ ^]^ Although such a method has achieved great success, it is generally not efficient. Site‐specific probes are limited to capturing only readers of the single site, so reader identification for numerous methylation sites by site‐specific probes would be quite time‐consuming and labor‐intensive. Therefore, a much more efficient way is highly desired to catch the rapidly growing knowledge of the methyllysine proteome.

Comprehensive proteomic profiling approaches may overcome these limitations. Activity‐based protein profiling (ABPP) is a chemoproteomic strategy that employs probes to specifically interact with a class of proteins sharing conserved functional features. ABPP has been applied to the investigation of enzymes in complex biological samples, providing a global view of the functional state of the proteome. A classic example is the development of ABPP probes containing a reactive fluorophosphonate group, which irreversibly binds the active site of serine hydrolases, leading to the discovery of previously uncharacterized serine hydrolases.^[^ ^35^ ^]^ Since then, a number of probes were developed, including vinyl sulfone probes for cysteine proteases, epoxide‐based probes for the papain family, electrophilic ketone probes for the caspase family, etc.^[^ ^36^, ^37^ ^]^ Moreover, ABPP also facilitates inhibitor discovery and screening as well as off‐target validations.^[^ ^38^, ^39^ ^]^ However, to the best of our knowledge, no such tools have been exploited for the profiling of lysine methylation readers. In this study, we aimed at broad profiling of methyllysine readers and developed a general probe that specifically binds to and crosslinks methyllysine readers without site‐specificity (Figure 1C). Such a general probe could eliminate the need for site‐specific design, achieve the identification of multiple methylation site readers, and the evaluation of selectivity of reader inhibitors. In this case, we are pushing the studies of readers from site‐by‐site probes to global profiling probes at a new level.

## 2 Results and Discussion

### 2.1 Prototype of Oligoglycine‐Based Dimethylsulfonium Peptides Crosslinks Diverse Methyllysine Readers

**Figure 2:**
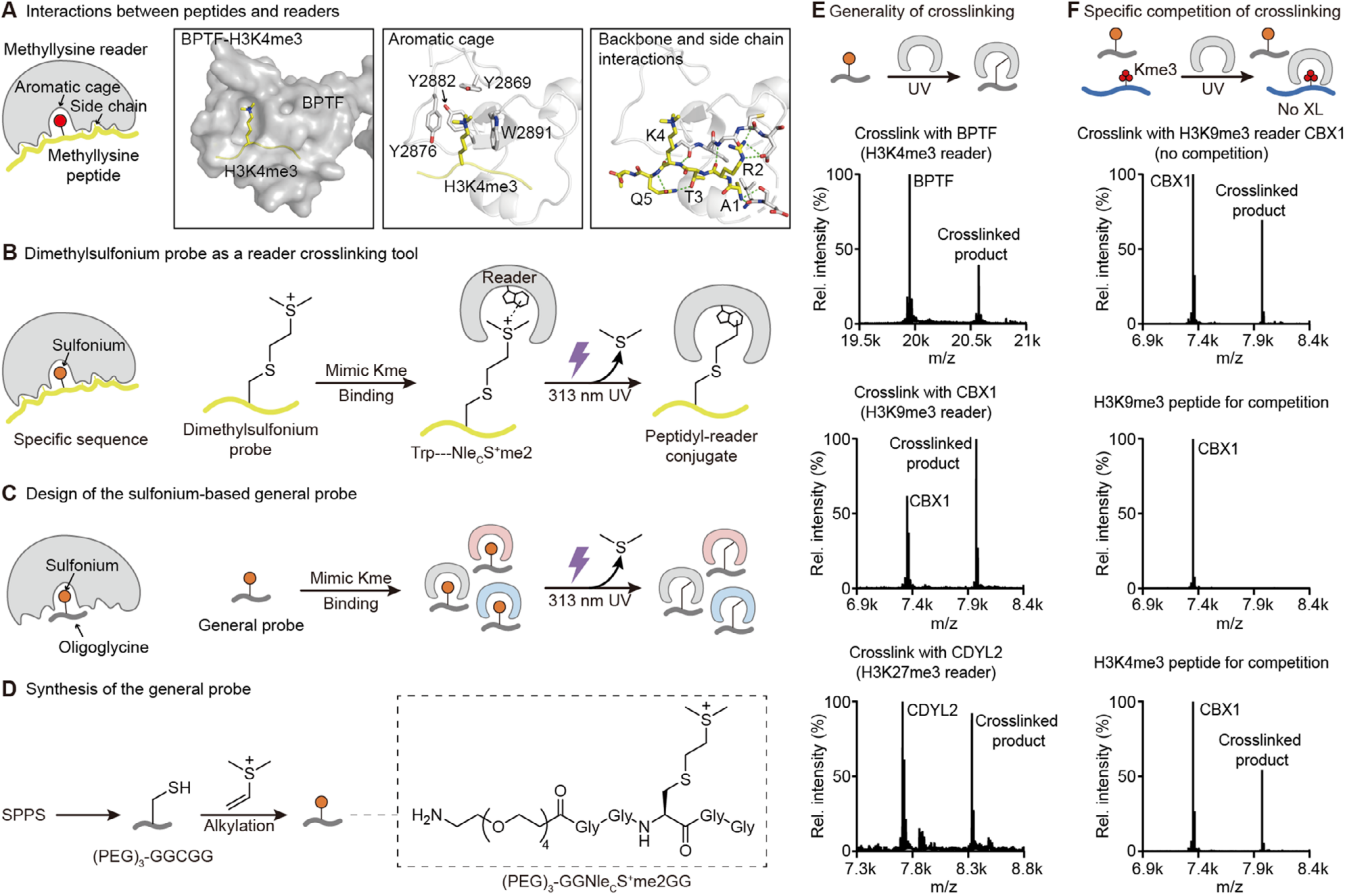
Design, synthesis, and proof of concept of oligoglycine‐based general probe for methyllysine reader crosslinking. A) Cartoon illustration of the general binding strategy of readers to methyllysine peptides and PyMOL structure (PDB 2F6J) of BPTF binding with H3K4me3 peptide as an example. In the PyMOL structure, from left to right, it highlights the surface and binding pocket of BPTF, interactions between the aromatic cage and the methyllysine, and interactions between the reader and the methylated peptide backbone and side chains. B) Reader binding‐dependent crosslinking strategy by dimethylsulfonium probes. Dimethylsulfonium mimics methyllysine, and the peptide sequence drives binding to specific readers for crosslinking to conserved tryptophan inside the aromatic cage via single‐electron transfer under UV irradiation. C) Design of the general probe for methyllysine readers. Dimethylsulfonium still provides interactions with the aromatic cage, while the sequence only offers backbone interactions without specific side chain interactions with readers. Therefore, the general probe can achieve binding and crosslinking to readers with different folding and site‐specificity. D) Synthetic route and structure of oligoglycine‐based general probe prototype. E) Crosslinking to different readers by the general probe and corresponding high‐resolution mass spectra of the reaction mixtures. F) Crosslinking of general probe with CBX1, competition by site‐specific methylated peptides, and high‐resolution mass spectra of the reaction mixtures.

A key premise of ABPP is a shared mechanism of the group of proteins. Generally, readers recognize methyllysine via conserved aromatic cages by cation‐π interactions and van der Waals forces, despite the different domains and folding.^[^ ^40^, ^41^, ^42^, ^43^, ^44^ ^]^ Taking BPTF as an example, its PHD domain created an aromatic cage with three tyrosines and a tryptophan for H3K4me3 binding (**Figure** **2**A).^[^ ^45^ ^]^ The general methyllysine binding strategy of readers presents an opportunity for global investigation by ABPP.

The next key challenge is the development of a warhead with general crosslinking activity to readers, because readers are not like enzymes that contain catalytically active residues or cofactors for crosslinking tool development. Sulfonium‐based tools have attracted increasing interest in chemical biology for protein conjugation and peptide/protein delivery due to their unique properties and reactivity.^[^ ^46^, ^47^, ^48^, ^49^, ^50^, ^51^, ^52^, ^53^ ^]^ Our group recently developed peptide and protein‐based sulfonium probes to selectively crosslink methyllysine readers (Figure 2B).^[^ ^54^, ^55^, ^56^, ^57^, ^58^, ^59^ ^]^ Dimethylsulfonium closely mimics the size and positive charge of dimethyllysine, facilitating its recognition by aromatic cages of reader binding pockets. The crosslinking is initiated by UV irradiation when dimethylsulfonium forms electron donor‐acceptor (EDA) complexes with conserved tryptophan inside the aromatic cage via probe‐reader binding, and the excited indole electron transfers to the sulfonium. After S─C bond homolysis with the release of dimethyl sulfide, the resulting carbon radical rebinds to the tryptophan cation radical to yield a selective conjugate. This binding‐mediated crosslinking ensures crosslinking specificity dependent on molecular recognition. Critically, these sulfonium probes demonstrate sufficient stability under physiological conditions, ensuring their integrity for biological applications. Later, the sulfonium tools were shown to selectively crosslink and identify diverse methyllysine readers from complicated cell samples. Therefore, dimethylsulfonium is an ideal warhead to develop general reader probes.

The third consideration is to design the probe without methyllysine site‐specificity. We went back to browse structural studies of readers and found that in addition to aromatic cages for methyllysine residue binding, readers interact with adjacent residues, including both side chains and backbone (Figure 2A). For example, BPTF interacts with Ala1, Arg2, Thr3, Lys4, and Gln5 by several hydrogen bonds and ionic interactions, which account for the histone H3 sequence. Therefore, we designed an oligoglycine‐based sulfonium peptide as a general probe for methyllysine readers (Figure 2C). The simplified oligoglycine residues still contain the backbone to maintain sequence‐independent interactions with readers, while their lack of side chains eliminates sequence‐specific recognition. Meanwhile, the dimethylsulfonium moiety is still able to crosslink tryptophan within the aromatic cages of readers.

We thus started with a prototype, NH_2_‐(PEG)_3_‐GGNle_C_S^+^me2GG (peptide **1**). The polyethylene glycol (PEG) serves as a linker for attachment of enrichment tags and enhancement of solubility in aqueous buffer. This designed probe was prepared by solid phase peptide synthesis (SPPS) followed by cysteine alkylation with dimethylvinylsulfonium (Figure 2D).^[^ ^55^ ^]^ 8.4 mg target probe was obtained with an overall 61% isolated yield. We subsequently tested the probe generality with different methyllysine readers, including H3K4me3 reader BPTF, H3K9me3 reader CBX1, and H3K27me3 reader CDYL2^[^ ^60^ ^]^ (Figure 2E). The reader and probe mixtures were irradiated with UV light, and the crosslinking reactions were analyzed by mass spectrometry. The data demonstrated that the probe was active to crosslink readers with different methyllysine site recognition and distinct domains, confirming its generality (Figure 2E). We also conducted a competitive assay with additional methyllysine peptides. The crosslinking with CBX1 was effectively competed by the H3K9me3 peptide but not the H3K4me3 peptide, indicating that the crosslinking by the probe depends on specific reader binding (Figure 2F). Hence, the prototype probe validated the concept of the sulfonium‐based general probe.

### 2.2 Improvement and Characterization of the Dimethylsulfonium‐Based General Probe

**Figure 3:**
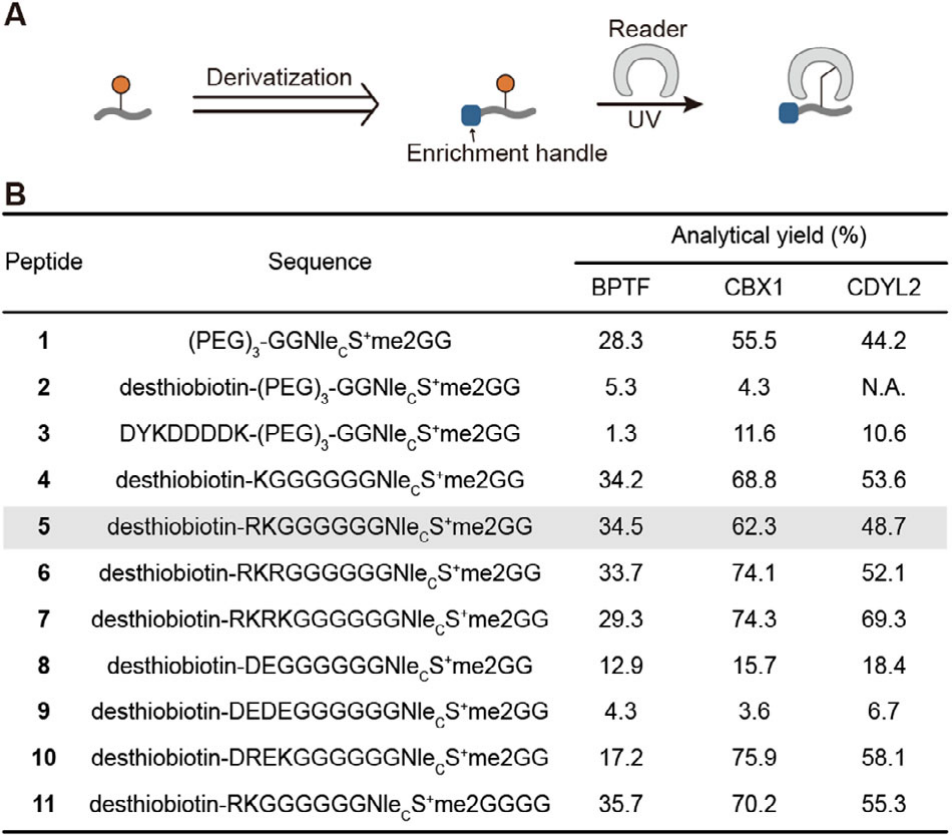
Optimization of the sulfonium‐based general probe. A) Different enrichment handles were introduced to the prototype of the general probe. The general probes were characterized by crosslinking. B) The table presents the analytical yields of general probes containing different charged amino acids and enrichment tags crosslinked with different readers, based on mass spectra. N.A.: not available.

Following the initial confirmation of the feasibility of the general probe, we further incorporated enrichment tags to adapt it for subsequent applications in complex samples (**Figure** **3**A). We first attempted to introduce the commonly used desthiobiotin and FLAG (DYKDDDDK) tag. However, the crosslinking reactivity of peptides **2** and **3** significantly decreased (Figure 3B). We hypothesized that desthiobiotin, which is highly hydrophobic, combined with the charged sulfonium of the probe, renders the entire probe a detergent‐like structure, thereby impairing crosslinking efficiency. Additionally, the FLAG tag contains multiple negatively charged aspartate residues, whereas the sulfonium group carries a positive charge, which suggests that intramolecular interactions may occur between these moieties, adversely affecting crosslinking.

Therefore, we thought the PEG was not polar enough and thus attempted to introduce charged amino acids into the probe, such as lysine, arginine, aspartate, and glutamate, aiming to further alleviate the hydrophobicity caused by desthiobiotin. Since the initially designed PEG linker was removed, we added more glycines between these amino acids and the sulfonium group to minimize potential interference from the functional side chains of these amino acids on the generality of the probe. These peptides were obtained at a 10 mg scale with an overall 20–55% isolated yield. Crosslinking experiments revealed that these new probes retained generality (Figure 3B; Figure S2, Supporting Information). The introduction of positive charges significantly improved the crosslinking efficiency, but the varying numbers of positively charged amino acids exerted little impact on crosslinking efficiency (peptides **4–7**). Compared to the positively charged probes, the probes carried negative charges, which are similar to the FLAG tag, exhibited decreased crosslinking efficiency, with a more obvious effect as the number of negative charges increased (peptides **8, 9**). For the probe with alternating positive and negative charges (peptide **10**), the crosslinking efficiency was comparable to that of probes carrying only positive charges. Moreover, increasing the number of glycine residues did not significantly enhance crosslinking efficiency (peptide **11**). Thus, we ultimately selected peptide **5** for subsequent experiments.

To further characterize peptide **5**, we performed kinetic analysis of the crosslinking process to CBX1 and BPTF (Figure S1, Supporting Information). Due to the lack of specific side‐chain interactions with readers, the binding affinity of the general probe was significantly weaker, as indicated by a higher apparent *K*_m_ value compared to that of specific probes.^[^ ^55^ ^]^ For example, the *K*_m_ of peptide **5** for BPTF was 0.43 ± 0.13 mM, whereas the *K*_m_ of the specific probe H3K4Nle_C_S^+^me2 for BPTF was 6.1 ± 1.3 µM. Meanwhile, the *k*_cat_ values for the general probe (0.013 ± 0.00074 min^−1^ for BPTF and 0.034 ± 0.0013 min^−1^ for CBX1) were lower than those of the specific probes (0.15 ± 0.0086 min^−1^ for BPTF and 0.20 ± 0.049 min^−1^ for CBX1), likely due to less efficient binding and consequently reduced stability of the EDA complex required for the single‐electron transfer between sulfonium and tryptophan. Based on the kinetic parameters, we set 2.5 mM as a reasonable concentration for the general probe to target diverse readers. Competitive experiments further confirmed that the crosslinking of this probe with readers could be specifically inhibited by competitive peptides (Figure S3, Supporting Information).

Since the sulfonium‐indole EDA complex is crucial for the crosslinking, wherein the sulfonium serves as an electron acceptor due to its electron‐deficient characteristics, we decided to characterize the electronic state of the sulfur by X‐ray photoelectron spectroscopy (XPS). To avoid potential interference from two sulfur atoms present in cysteine‐alkylated sulfonium peptides, we synthesized methionine‐based sulfide peptide **S4’** as well as the corresponding sulfonium peptide **4’** via iodomethane alkylation of methionine for XPS analysis. The sulfide peptide displayed the expected S 2p binding energy for a thioether signal (S 2p3/2 ≈163.2 eV, S 2p1/2 ≈164.4 eV). In contrast, the S 2p spectrum of sulfonium peptide **4’** exhibited a higher binding energy consistent with reported values^[^ ^61^, ^62^ ^]^ for sulfonium cations (S 2p3/2 ≈165.7 eV, S 2p1/2 ≈166.9 eV) (Figure S4, Supporting Information). The coexistence of a thioether signal may be attributed to partial conversion from the sulfonium due to potential beam damage under the high‐energy X‐ray radiation during XPS measurement. These results provide physical evidence for the presence of the key sulfonium center as more electron‐deficient than sulfide, corroborating its role as an electron acceptor in the EDA complex.

Collectively, the kinetic study, competitive crosslinking, and physicochemical characterization demonstrated that the general probe can specifically recognize and crosslink multiple methyllysine readers. The specificity and mechanistic insight qualified our improved probe for applications in complicated cell samples.

### 2.3 The Sulfonium General Probe Selectively Crosslinked Cell Nuclear Readers by Methyllysine Peptide Competition

**Figure 4:**
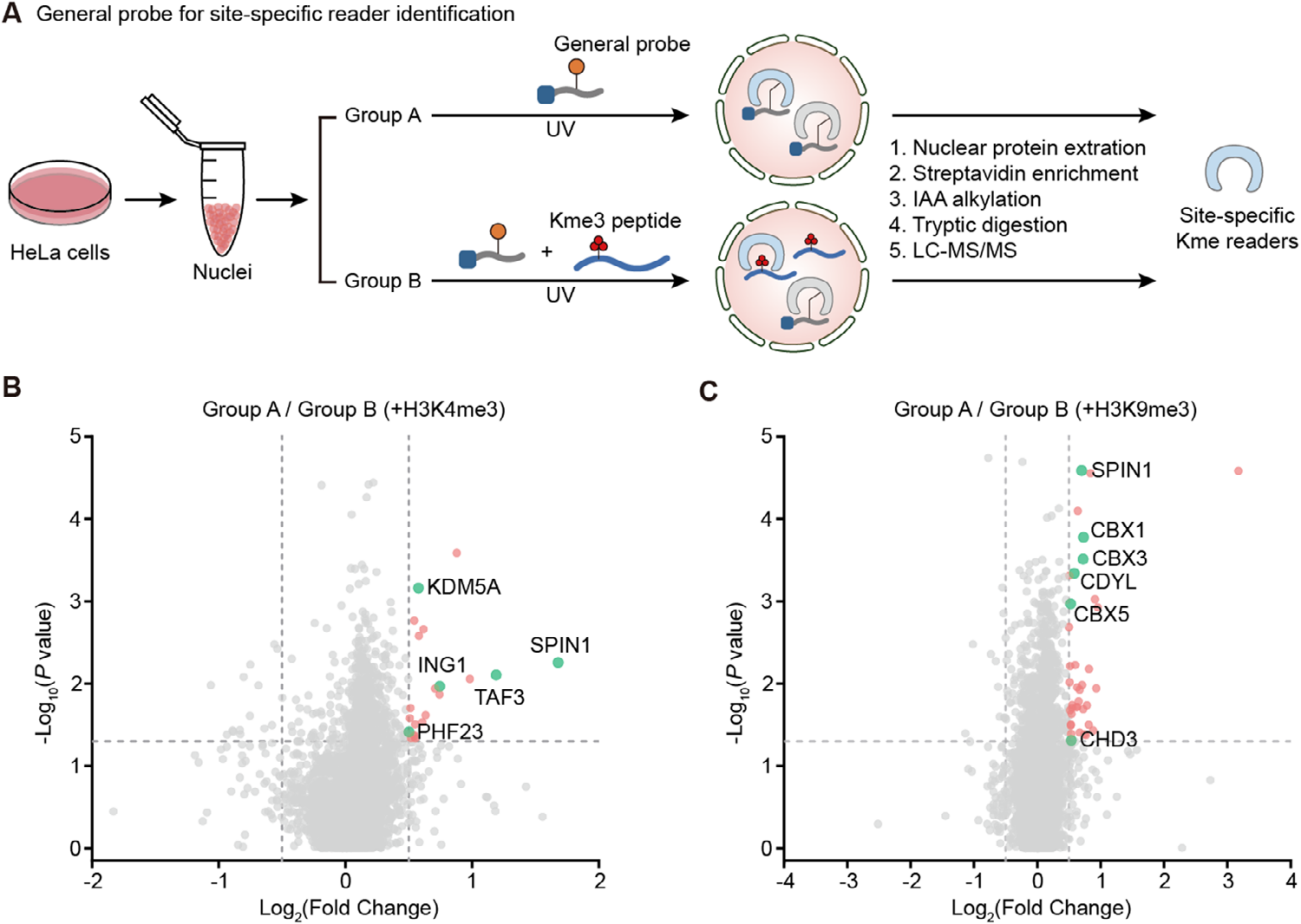
General probe for site‐specific reader crosslinking in nuclear proteins by methylated peptide competition. A) Schematic illustration of site‐specific reader investigation by a general probe. Extracted HeLa cell nuclei were treated with 2.5 mM general probe **5**. Group B was treated with an extra 200 µM methylated peptide. After 313 nm UV irradiation for 20 min, crosslinked proteins were enriched and digested for LC‐MS/MS analysis. B,C) Volcano plots of the proteomic experiment competed by H3K4me3 (B) or H3K9me3 (C). The hits for known H3K4me3 (B) or H3K9me3 (C) readers with Log_2_(Fold Change) ≥0.5 and *p* value ≤ 0.05 are shown as green dots with their name.

One advantage of the general probe is to apply the same probe to profile diverse methyllysine site readers by methyllysine peptide competition. This concept has been proven by in vitro assay, but the potential in cell samples is uncertain. We thus selected a well‐studied methylation site, histone H3K4, for proteomic exploration by methyllysine peptide competition. HeLa cell nuclei were extracted and divided into two groups: one group was incubated with 2.5 mM general probe (peptide **5**), while the other group was incubated with peptide **5** and an additional 200 µM H3K4me3 peptide. After UV irradiation, the nuclear proteins were extracted, and the crosslinked proteins were enriched by streptavidin beads via desthiobiotin tag of the probe and digested by trypsin for LC‐MS/MS analysis (**Figure** **4**A). Volcano plot from proteomic data revealed that 21 proteins were significantly enriched (Log_2_(Fold Change) ≥0.5, *p* value≤ 0.05), including 5 known H3K4me3 readers (Figure 4B). Encouraged by the successful validation at the H3K4 site, we also applied the same experimental workflow to the H3K9 site by usage of H3K9me3 peptide. The volcano plot showed significant enrichment of 38 total proteins, among which 6 hits were previously reported H3K9me3 readers (Figure 4C).

These results demonstrated that when paired with site‐specific methylated competing peptides, the general probe can identify potential readers specific to the site, providing support for the utility of the strategy for reader identification at specific methylation sites. In consequence, synthesis of each individual site‐specific sulfonium probe is not inevitable, and new reader discovery by general probe‐based screening could be much more efficient than the previous method.

### 2.4 The Sulfonium General Probe can be Applied to Analyze Methyllysine Inhibitor Activity and Selectivity

**Figure 5:**
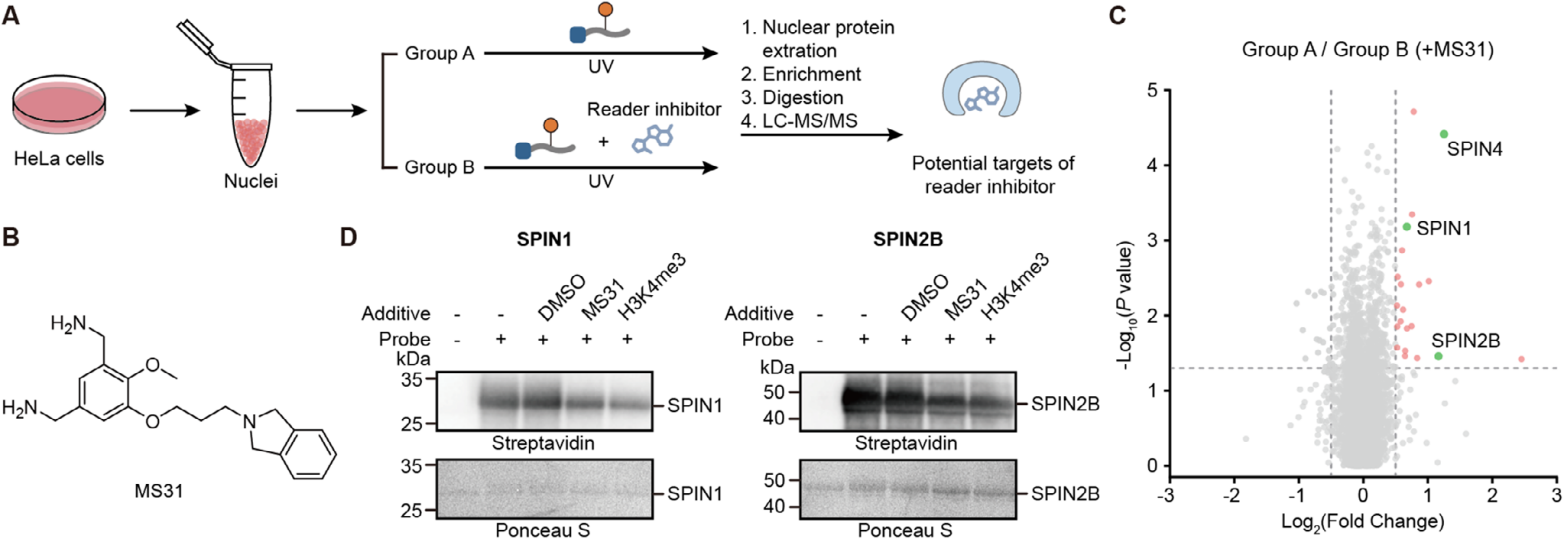
General probe for evaluation of reader inhibitor activity and selectivity in nuclear proteins by inhibitor competition. A) Schematic illustration of inhibitor assessment by general probe **12**. The workflow is similar to site‐specific reader investigation, while 200 µM MS31 was added to Group B rather than methylated peptides. B) Structure of MS31, an inhibitor of SPIN1 for H3K4me3 binding. C) Volcano plot of the proteomic experiment. The hits that have been reported capable of binding with MS31 with Log_2_(Fold Change) ≥0.5 and *p* value ≤ 0.05 are shown as green dots with their name. D) Western blot analysis of MS31 activity to SPIN1 and SPIN2B by probe **12**.

Given the capacity of the general probe for specific methyllysine reader identification by methyllysine peptide competition, we may expand the general probe for evaluation of inhibitor selectivity, inspired by serine protease inhibitor screening by ABPP.^[^ ^63^ ^]^ Although some reader inhibitors have entered preclinical or clinical stages,^[^ ^20^ ^]^ the selectivity of many inhibitors remains to be thoroughly investigated. The off‐target risks of inhibitors potentially arising from commonalities of binding mode among methyllysine readers. Present studies often rely on in vitro single‐target experiments, but the coverage of readers is rather limited from recombinant expression, making it difficult to comprehensively cover potential targets. In addition, in vitro experiments may not reflect the activity in cells. We hypothesized that the general probe could help to find the potential targets of the inhibitor, a strategy similar to that used in site‐specific reader identification (**Figure** **5**A). In the presence of an inhibitor, competitive binding would reduce the enrichment of its specific targets (both known and unknown) in proteomic analyses, enabling systematic assessment of inhibitor selectivity.

To verify this approach, we selected MS31, a potent and selective fragment‐like inhibitor that primarily targets the H3K4me3 reader SPIN1 by binding to its Tudor2 domain.^[^ ^64^, ^65^, ^66^ ^]^ Besides its high affinity for SPIN1 (*K*_d_ 91 nM), MS31 has been reported to exhibit moderate binding affinity to other SPIN family members, including SPIN2B and SPIN4, while maintaining high selectivity over a broad panel of other epigenetic readers and writers.^[^ ^64^ ^]^ We used peptide **12**, which replaced desthiobiotin of peptide **5** with biotin to enhance binding affinity to streptavidin beads, for a proteomic experiment by the same procedure of histone Kme3 peptide competition (Figure 5B). The results revealed that 21 proteins were significantly enriched, among which the expected primary target SPIN1 was identified, along with two other SPIN family members, SPIN4 and SPIN2B, which are known to bind MS31 with moderate affinity (Figure 5C). We also confirmed in vitro that the crosslinking between SPIN2B and probe **12** can be inhibited by MS31 (Figure 5D).

These findings highlight the potential of the general probe to uncover both on‐target and previously unrecognized interactions of inhibitors, offering a more comprehensive view of their selectivity profiles in cells than traditional single‐target validation using recombinant readers. Moving forward, this strategy could facilitate rational optimization of inhibitors by prioritizing specificity toward intended targets, while also expanding our understanding of inhibitor‐mediated regulatory networks in complex biological systems.

### 2.5 Exploration of Methyllysine Reader Proteome by the Sulfonium General Probe

**Figure 6:**
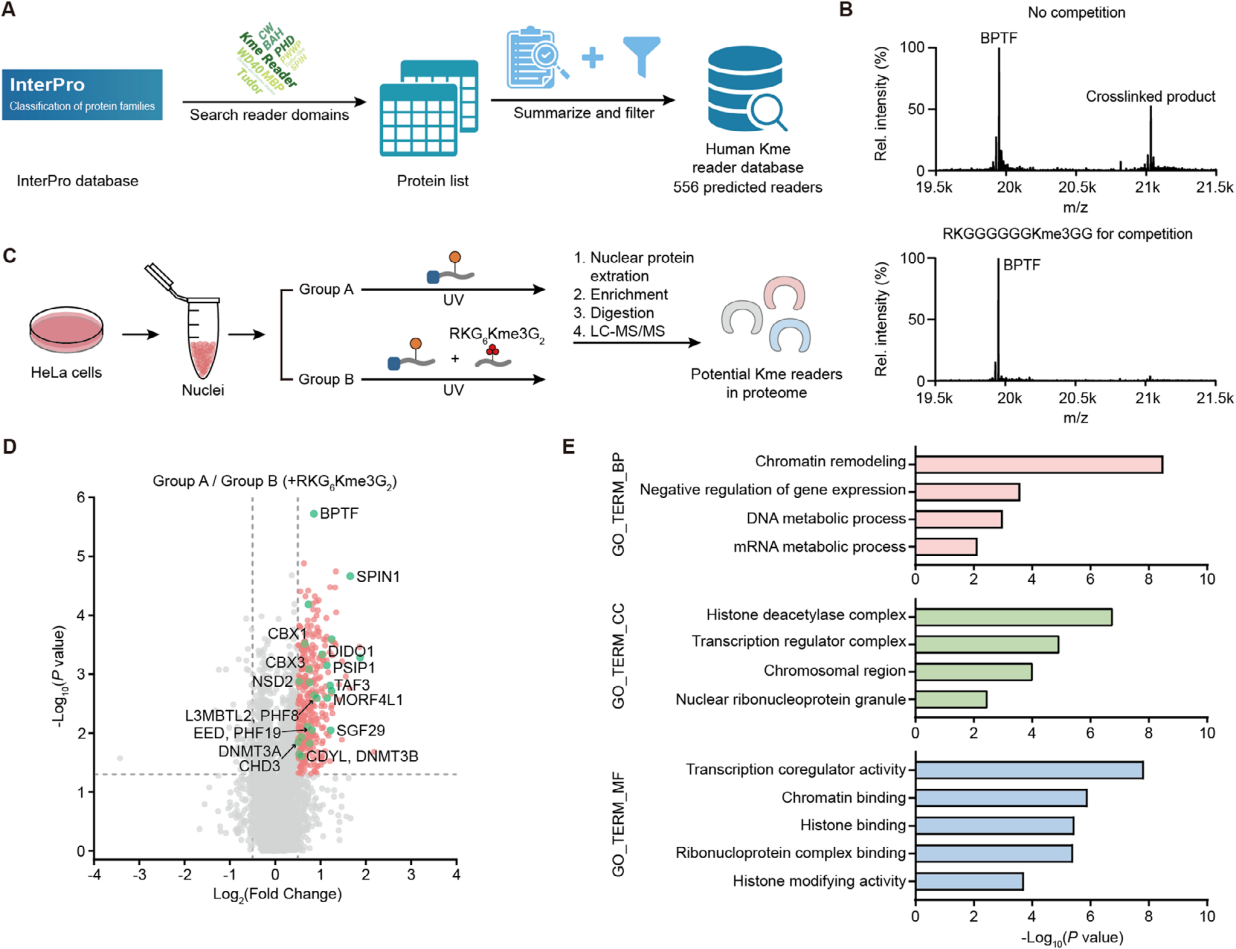
General probe for global profiling of readers. A) Bioinformatic analysis defining the landscape of potential methyllysine readers in the human genome by the InterPro database. B) In vitro crosslinking validation of the competitive effect of the general competition peptide (RKGGGGGGKme3GG, peptide **13**). C) Schematic illustration of global profiling of readers by general probe **12**. Extracted HeLa cell nuclei were treated with 2.5 mM general probe. Group B was treated with probe **12** and an extra 7.5 mM peptide **13**. The samples were crosslinked, enriched, and digested for LC‐MS/MS analysis. D) Volcano plot of the proteomic experiments. The hits for known readers with Log_2_(Fold Change) ≥0.5 and *p* value ≤ 0.05 are shown as green dots with their names. (E) Gene ontology analysis of 22 unreported potential readers containing classical reader domains by Metascape. BP: biological process, CC: cellular component, MF: molecular function.

The utility of the general probe for methyllysine peptide and inhibitor competition demonstrated the broad crosslinking activity to readers in cell samples. Hence, we aimed to push forward with systematic profiling in the proteome. To establish a reference framework for this exploration, we first conducted a bioinformatic analysis to define the landscape of potential methyllysine readers in the human genome (**Figure** **6**A). Using the InterPro database,^[^ ^67^ ^]^ we systematically retrieved proteins encoded by the human genome (Homo sapiens, taxon ID: 9606) that contain known or putative methyllysine‐binding domains, including 13 InterPro entries corresponding to domains such as PWWP, chromodomain, BAH, Tudor, and PHD fingers. After removing redundancies and splice variants to retain only the principal isoform of each gene, we constructed a comprehensive dataset of predicted human methyllysine reader candidates (Spreadsheet S1, Supplementary file), which includes the gene symbol, Uniprot accessions, and the classical domains potentially contained in the proteins. The dataset revealed ≈588 candidates. Among these, 32 proteins contained two reader domains, resulting in 556 unique candidate proteins. This distribution indicated that the vast majority of predicted readers only contained a single reader domain. This number is far more than known methyllysine readers, which indicates indeed the potential to explore new readers.

Since we focus on global profiling, a nonspecific competitor would be required rather than site‐specific Kme3 peptide and small molecule inhibitors. We first synthesized a competing peptide (peptide **13**, RKGGGGGGKme3GG), which shares the same sequence as the probe but lacks the enrichment tag, contains a trimethyllysine residue instead of the sulfonium group, and is intended to competitively block binding sites on proteins with methyllysine recognition activity. Crosslinking assays in vitro validated that three equivalents of the peptide were sufficient to achieve complete competition (Figure 6B). Based on this, HeLa cell nuclei were divided into two groups: one incubated with 2.5 mM general probe alone, and the other co‐incubated with both the general probe and 7.5 mM general competing peptide in the proteomic experiment. By comparing the two groups, proteins specifically enriched in the probe‐only group would be considered candidates with methyllysine‐binding potential (Figure 6C).

Proteomic analysis revealed 379 proteins significantly enriched in the probe‐only group (Figure 6D). Among these, 46 proteins overlapped with our bioinformatically defined dataset, indicating they contain classical methyllysine‐recognizing modules (Table S1, Supporting Information). Within these 46, 24 hits appear to be reported readers, including those targeting H3K4me3, H3K9me3, H3K27me3, and H3K36me3, demonstrating the ability of the general probe to capture readers across diverse methylation sites. Further analysis confirmed the breadth of coverage: the 46 proteins contained major reader domains such as WD40 repeats, PHD fingers, CW domains, Tudor domains, BAH domains, PWWP domains, chromodomains, and MBT domains, reflecting the versatility of the general probe across distinct structural classes of readers.

Next, we were curious about the 22 unreported potential new readers captured by the general probe. To evaluate the function of the 22 candidates, we performed gene ontology (GO) analysis in Metascape^[^ ^68^ ^]^ encompassing three groups: the 22 unreported putative readers, the 24 known readers captured in our profiling, and the entire bioinformatic dataset of 556 predicted readers. The GO analysis revealed a functional alignment of the 22 candidates with 24 established readers (Figure 6E; Figure S5, Supporting Information). Both groups were significantly enriched in core epigenetic processes, such as chromatin remodeling and negative regulation of gene expression, as well as molecular functions including chromatin binding. Cellular components of 22 potential readers highlighted localization to the histone deacetylase complex and transcription regulator complex, suggesting potential crosstalk between lysine methylation and acetylation. The functional similarity suggests that the unreported candidates are likely to be methyllysine readers. The GO analysis of 556 predicted proteins with reader domains revealed significant enrichment of terms related to epigenetic regulation. However, the functional spectrum was broader (Figure S6, Supporting Information). It may not only reflect the diverse functional potential of proteins but also indicate that the general probe‐based activity profiling selectively enriched a functionally coherent subset of readers.

We also compared the significantly enriched proteins from both the H3K4me3 and H3K9me3 experiments with our bioinformatically predicted database of 556 predicted proteins. Notably, no other significant hits matched the predicted reader database except reported readers. This observation can be attributed to several factors common in proteomic workflows, such as non‐specific binding or the indirect capture of protein complexes. Crucially, the competitive profiling strategy successfully enriched site‐specific readers above the background.

These results suggest that the general probe can serve as a useful tool for unbiased profiling of methyllysine readers, offering an alternative to site‐specific approaches. By capturing both known readers with broad site specificities and uncharacterized domain‐containing proteins, it contributes to efforts to expand the methyllysine interactome, particularly for understudied non‐histone substrates, and provides a basis for investigating the functional roles of novel readers in epigenetic regulation.

## 3 Conclusion

In summary, this study presents a conceptual innovation in chemical tools for investigating methylysine readers. Moving beyond the previous paradigm of designing site‐specific probes for one methylation site, we designed and synthesized general probes based on a dimethylsulfonium warhead and oligo‐glycine peptides as the scaffold, aiming to systematically investigate lysine methylation readers. Through characterization of crosslinking activities, we confirmed that this general probe can bind and crosslink various methylation readers without being restricted by methyllysine site or reader domain. We have demonstrated the feasibility of the general probe as a versatile tool for multiple applications. When coupled with quantitative proteomics and competitive strategies, it allows for the identification of site‐specific readers by competing with site‐specific methylated peptides. It assists in evaluating inhibitor selectivity in cellular contexts. Furthermore, the probe facilitates unbiased global profiling of methyllysine readers, capturing 46 candidates containing classical reader domains. These results indicate that this general probe provides a practical tool for broad‐spectrum identification and systematic exploration of methylation readers, particularly holding promise for underexplored areas such as non‐histone readers outside of the nucleus.

To the best of our knowledge, this represents the first chemical probe strategy for global profiling of methyllysine readers. A previous study by the Kutateladze lab reported a rapid structural motif‐mining algorithm, *Erebus*.^[^ ^69^ ^]^ This algorithm predicts potential readers based on geometric or structural similarity. However, it relies on static structural information and cannot directly validate the binding activity of potential readers. In contrast, our activity‐based profiling method directly reports the binding activity of putative readers within complex proteomes. We envision that such a general probe will serve as a systematic mapping tool for the methyllysine reader landscape.

## 4 Experimental Section

Essential Experimental Procedures/Data. ((All other characterization data, original spectra, etc., should be provided in the Supporting Information)).

### Materials and Reagents

Chemicals used in this work including all fluorenyl methoxycarbonyl (Fmoc) amino acids, Fmoc‐Lys(me3)‐OH, Rink Amide‐AM Resin, desthiobiotin, biotin, *N,N*‐dimethylformamide (DMF), *N*‐methyl‐2‐pyrrolidone (NMP), *N,N’*‐diisopropylcarbodiimide (DIC), ethyl cyanoglyoxylate‐2‐oxime (oxyma), (HCTU), *N,N*‐Diisopropylethylamine (DIPEA), 4‐methylpiperidine, trifluoroacetic acid (TFA, HPLC grade), phenol, triisopropylsilane(TIPS), dimethyl ether, acetonitrile (ACN, HPLC grade), methanol (MeOH), dichloromethane(DCM), NH_2_‐(PEG)_3_‐COOH, methionine, dithiothreitol (DTT), formic acid (HCOOH), iodomethane (MeI), silver trifluoromethanesulfonate (AgOTf), acetic acid, isopropanol were purchased from GL Biochem, Bidepharm, Adamas, J&K Scientific, Meryer, Macklin, Sinopharm and VWR Life Science. Sodium dihydrogen phosphate (NaH_2_PO_4_), sodium hydrogen phosphate (Na_2_HPO_4_), 4‐(2‐hydroxyethyl)piperazine‐1‐ethanesulfonic acid (HEPES), guanidine hydrochloride (Gdn·HCl), tris (hydroxymethyl) aminomethane (Tris), sodium chloride (NaCl), potassium chloride (KCl), magnesium chloride (MgCl_2_), Nonidet P 40 (NP40), glycerol, bovine serum albumin (BSA), iodoacetamide (IAA), ammonium bicarbonate (NH_4_HCO_3_) were purchased from Titan, Solarbio, BBi Life Sciences Corporation, Sinopharm, Beyotime and Sigma–Aldrich.

### HPLC and Mass Spectrometry

Peptides used in this work were purified by reverse‐phase high‐performance liquid chromatography (RP‐HPLC) (Waters, 1525 binary pump and 2489 UV–vis detector) equipped with the C18 column (XBridge, Peptide BEH C18 column, 130 Å, 5 µm, 19 mm × 150 mm). The flow rate of the mobile phase was 10 mL min^−1^. The mobile phase A is H_2_O (0.1% TFA), and the mobile phase B is ACN (0.1% TFA). The peptides were detected by UV absorbance at 220 and 238 nm. Peptides were characterized by Matrix‐Assisted Laser Desorption/Ionization Time of Flight Mass Spectrometry (MALDI‐TOF) (Bruker, Rapiflex) matrixed with α‐cyano‐4‐hydroxycinnamic acid (HCCA). Peptides with sulfonium groups were analyzed by electrospray ionization‐time of flight mass spectrometry (ESI‐TOF) (Waters, Synapt XS HDMS [high definition MS system]).

### General Procedure for Peptide Synthesis

Peptides were synthesized based on standard fluorenyl methoxycarbonyl‐solid‐phase peptide synthesis (Fmoc‐SPPS) using a Liberty Blue 2.0 automated microwave peptide synthesizer (CEM Corporation, U.S.A.). Initially, 0.1 mmol Rink Amide‐AM resin was swollen in DMF for 10 min. The synthesis of peptides followed the standard deprotection and coupling cycles.

1. **Deprotection**: 4 mL of 4‐methylpiperidine (20% in DMF) was added to the reaction vessel. The mixture was maintained at 90 °C for 1 min under N_2_ bubbling, followed by four times washing with 5 mL DMF.
2. **Coupling**: 2.5 mL of 0.2 M Fmoc‐AA‐OH in DMF (5 equiv.), 1 mL of 0.5 M DIC in DMF (5 equiv.), and 1 mL of 1 M oxyma in DMF (10 equiv.) were added to the reaction vessel. The reaction was maintained at 90 °C for 2 min. For peptides with desthiobiotin or biotin tags, the resin that had finished the final deprotection was transferred to a peptide reaction tube to achieve the final coupling of desthiobiotin or biotin. The resin was washed with DMF three times, followed by the addition of 4 mL NMP solution containing 0.5 mmol desthiobiotin/biotin (5 equiv.), 0.5 mmol HCTU (5 equiv.), 1.0 mmol DIPEA (10 equiv.) for 1–1.5 h rotation at room temperature.
3. **Cleavage**: The resin was transferred to a peptide reaction tube with DMF, washed three times with DMF, DCM, and MeOH in sequence, and drained to be sandy. 4 mL cleavage cocktail (TFA:phenol:TIPS:H_2_O = 88:5:2:5) was added for 3 h rotation at room temperature. Finally, the crudes were precipitated and washed with ice‐cooled dimethyl ether.
4. **Purification**: The dried crude was dissolved in H_2_O (0.1% TFA) and injected into RP‐HPLC equipped with a C18 column and purified with a gradient of 5–30% B. The correctly identified peptides by MS were collected, evaporated, lyophilized, and stored at −20 °C until usage. H3K4me3 (1‐15) and H3K9me3 (1‐15) peptides were from previous work.^[^ ^54^ ^]^

### General Procedure for Peptide Alkylation

Peptides were alkylated as previously reported.^[^ ^55^ ^]^ Briefly, 5 mM peptides were added to the alkylation buffer A (200 mM NaH_2_PO_4_‐Na_2_HPO_4_, 4 M Gdn·HCl, 10 mM Met, pH 7.3), followed by the addition of 10 mM DTT. The mixture was incubated at 37 °C for 1 h. Then, the tubes were placed on ice for 10 min. Next, 1 M dimethlvinylsulfonium (synthesized as published^[^ ^55^ ^]^) in NMP was added (final concentration: 50 mM), and the reaction was proceeded at 37 °C for another hour. Peptide **S4’** was alkylated following a previously reported procedure^[^ ^70^ ^]^ to yield peptide **4’**. Briefly, 5 mM peptide was added to the alkylation buffer B (0.4 M HCOOH/ACN = 1:1, v/v), followed by the addition of 100 mM iodomethane. Subsequently,100 mM AgOTf was added, and the reaction was processed in a ThermoMixer at 37 °C for 16 h. Finally, these peptides were purified by RP‐HPLC and characterized by ESI‐MS. The target peptides were collected, evaporated, lyophilized, and stored at −20°C.

### X‐Ray Photoelectron Spectroscopy

X‐ray photoelectron spectroscopy (XPS) was performed by PHI Genesis (ULVAC‐PHI) with a monochromatic Al Kα X‐ray source with the X‐ray beam size of 100 µm. The samples (peptide **S4’** and **4’**) were prepared by dusting the solid powder onto a double‐sided adhesive carbon tape mounted on a standard stainless steel sample stub. Charge compensation was achieved by the dual beam charge neutralization, and the binding energy was corrected by setting the binding energy of the hydrocarbon C 1s feature to 284.8 eV. Data analysis and peak fitting of the S 2p region were carried out using Multipak software. The S 2p spectra were deconvoluted using a Shirley background and a mixed Gaussian–Lorentzian, with the S 2p3/2 and S 2p1/2 doublet constrained to a spin‐orbit splitting of 1.18 eV, an area ratio of 2:1, and an equal full width at half maxima (FWHM).

### Expression and Purification of Proteins

BPTF, CBX1, and SPIN1 were expressed and purified as previously reported.^[^ ^54^, ^59^ ^]^ Full‐length SPIN2B (residues 1–258) from human was cloned into the pET‐28a vector with an *N*‐terminal 6×His‐SUMO‐tag. It was over‐expressed in Rosetta (DE3) *Escherichia coli* cells by induction of 0.25 mM isopropyl β‐D‐thiogalactoside (IPTG) at 16 °C overnight when OD600 reached 0.6–0.8 in the LB medium. The harvested cells were suspended in lysis buffer (20 mM Tris, 150 mM NaCl, 0.2 mM PMSF, pH 7.5) and lysed by sonication; then, the cell lysate was centrifuged to remove the insoluble cell debris. The clarified supernatant was loaded on nickel resin equilibrated in lysis buffer. Afterward, the protein‐bound resin was washed sequentially with lysis buffer, high salt buffer (20 mM Tris, 500 mM NaCl, pH 7.5), and 20 mM imidazole buffer (20 mM Tris, 150 mM NaCl, 20 mM imidazole, pH 7.5). The target proteins were eluted by 200 mM imidazole buffer (20 mM Tris, 150 mM NaCl, 200 mM imidazole, pH 7.5), followed by the solution exchanged to storage buffer (20 mM Tris, 150 mM NaCl, pH 7.5). The product was concentrated, snap‐frozen, and stored at −80°C. The gene encoding the chromodomain of human CDYL2 corresponding to residues 2–64 was inserted into the pET‐28a vector with an *N*‐terminal 6×His‐SUMO‐tag. Rosetta (DE3) *E. coli* cells transformed with the expression plasmid were cultured at 37 °C with shaking and then added to 0.2 mM IPTG when OD600 reached 0.6–0.8. The cells were induced to overexpress target proteins at 16 °C for 15 h. The harvested cells were suspended in lysis buffer (20 mM Tris, 150 mM NaCl, 0.2 mM PMSF, pH 7.5) and then lysed through sonication. The supernatant after centrifugation was loaded on nickel resin equilibrated in lysis buffer. The resin was washed sequentially by lysis buffer, high salt buffer (20 mM Tris, 500 mM NaCl, pH 7.5), and 20 mM imidazole buffer (20 mM Tris, 150 mM NaCl, 20 mM imidazole, pH 7.5). Target proteins were eluted by 200 mM imidazole buffer (20 mM Tris, 150 mM NaCl, 200 mM imidazole, pH 7.5) and then treated by Ulp1 from previous work^[^ ^32^ ^]^ to remove the 6×His‐SUMO tag. Purified proteins were separated by nickel resin again. Finally, the flow‐through solution was dialyzed into the storage buffer (20 mM Tris, 150 mM NaCl, pH 7.5) and concentrated. The proteins were snap‐frozen and stored at −80°C.

### General Procedure for Crosslinking

Probes were added to the crosslinking buffer (100 mM HEPES, pH 7.5), followed by the addition of reader proteins. The mixture was transferred to 96‐well plates and incubated on ice for 10 min. After UV irradiation on ice at an energy of 720 mJ cm^−2^, the samples were analyzed by UPLC‐Q‐TOF‐MS. The analytical yield was calculated based on the mass peak areas as previously reported.^[^ ^55^ ^]^ The yield is calculated as the peak area of the desired product divided by the sum of the peak areas of the crosslinking products and the starting material, multiplied by 100%. For crosslinking readers with general probes: The mixture of 5 mM general probes and 10 µM reader proteins (BPTF, CBX1, CDYL2) was crosslinked by the *General procedure for crosslinking* for 30 min. For competition assays, the mixture of 2.5 mM general probes, 100 µM H3K4me3 (1‐15) or H3K9me3 (1‐15) peptide or 7.5 mM RKGGGGGGKme3GG, 10 µM reader proteins (BPTF, CBX1) was crosslinked by the *General procedure for crosslinking* for 20 min (H3K4me3 or H3K9me3 for competition) or 30 min (RKGGGGGGKme3GG for competition). For Kinetics of reader crosslinking: The mixture of 0.625/1.25/2.5/5/10 mM general probe and 10 µM reader proteins was crosslinked by the *General procedure for crosslinking* for 20 min. Based on the analytical yields, the data were processed using GraphPad 8.0 and the *K*_m_ and *k*_cat_ values through the fitted Michaelis–Menten equation curve.

### Cell Lines and Cell Culture

HeLa cells, procured from ATCC, were cultured in Dulbecco's modified Eagle's medium (DMEM, Gibco, 11995500BT) supplemented with 10% fetal bovine serum (FBS, CellMax, SA211.02), 100 U mL^−1^ penicillin, and 100 µg mL^−1^ streptomycin. The cells were maintained in a humidified atmosphere at 37 °C with 5% CO_2_.

### General Procedure for Proteomic Experiment

HeLa cells (1.8 × 10^7^) were harvested, washed with pre‐cooled phosphate buffer saline (PBS), and lysed in hypotonic buffer (10 mM Tris, 15 mM NaCl, 1.5 mM MgCl_2_, 0.2 mM PMSF, pH 7.5) on ice with regular inversion for 10–15 min. After centrifugation at 200 g for 5 min at 4°C, the pellet was resuspended in hypotonic buffer and homogenized with three strokes of a loose pestle Dounce homogenizer. The intact nuclei were washed with a hypotonic buffer and collected by centrifugation. Then, the nuclei were resuspended in crosslink buffer (100 mM HEPES, 150 mM KCl, 1.5 mM MgCl_2_, 0.2 mM PMSF, pH 7.5) and divided into two groups (Group A and Group B), each of which contained three parallel samples. The general probe was added to Group A. The same amount of the general probe and extra competing peptides was added to Group B. These samples were transferred to a 24‐well plate, incubated on ice for 10 min, and irradiated under 313 nm UV for 20 min on ice. Next, the nuclei were washed with crosslinking buffer three times. Subsequently, the nuclear proteins were obtained by sonication in nuclear extraction buffer (50 mM HEPES, 300 mM KCl, 1.5 mM MgCl_2_, 0.1% NP40, 10% glycerol,.0.2 mM PMSF, pH 7.5). After centrifugation at 12000 rpm for 5 min, the supernatant was loaded onto Pierce streptavidin magnetic beads (Thermo Fisher, 88817), which were equilibrated and blocked with nuclear extraction buffer containing 5% BSA. After 3 h rotation at 4°C, the beads were washed with wash buffer (50 mM HEPES, 150 mM KCl, 1.5 mM MgCl_2_, 5% glycerol, pH 7.5) twice and 50 mM NH_4_HCO_3_ twice. Then, the beads were treated with 10 mM DTT in 50 mM NH_4_HCO_3_ in a ThermoMixer at 37 °C at 1000 rpm for 1 h. Next, 20 mM IAA was added, and the samples were placed in the ThermoMixer at 25 °C at 1000 rpm for 30 min in the dark. Subsequently, the beads were washed with 50 mM NH_4_HCO_3_ four times and resuspended in 200 µL 50 mM NH_4_HCO_3_. After that, 10 ng µL^−1^ Gold Trypsin was added for on‐beads digestion in a ThermoMixer at 37 °C at 1500 rpm for 12 h. An extra 5 ng µL^−1^ Gold Trypsin was added for another 2 h of digestion. Finally, the supernatant was collected, and the beads were washed with 200 µL 50 mM NH_4_HCO_3_. The combined supernatant was centrifuged at 12 000 rpm for 5 min and lyophilized for further LC‐MS/MS.

For LC‐MS/MS analysis, peptide separation was achieved using a 130 min gradient elution at a flow rate of 0.300 µL min^−1^, employing a Thermo Vanquish Neo integrated nano‐HPLC system that was directly coupled to a Thermo Exploris 480 mass spectrometer. The analytical column used was a home‐made fused silica capillary column (75 µm ID, 250 mm length; Upchurch, Oak Harbor, WA) packed with C‐18 reversed‐phase resin (1.7 µm; Varian, Lexington, MA). Mobile phase A was composed of 0.1% formic acid in water, while mobile phase B comprised 80% acetonitrile with 0.1% formic acid. The elution gradient was programmed as follows: starting at 1% B, it was increased to 23% B over 88.3 min, then raised to 40% B by 116.3 min, further increased to 65% B at 117 min, and maintained at 99% B from 119 to 130 min. The mass spectrometer was operated in a FAIMS data‐dependent acquisition mode (CV = −45, −65) via Xcalibur 4.5 software. Each acquisition cycle included a single full‐scan mass spectrum in the Orbitrap (350–1800 m/z range, 60 000 resolution), followed by MS/MS scans (15 000 resolution) with a 1 s cycle time and 30% normalized collision energy.

Label‐free quantification was performed with Proteome Discoverer 2.5, which was used for database searches against the Homo sapiens (Human) proteome database retrieved from UniProtKB (accession UP000005640). The search parameters for Sequest were set as follows: a precursor mass tolerance of 10 ppm, a fragment ion tolerance of 0.02 Da, and a maximum of 2 internal cleavage sites. Fixed modifications comprised cysteine alkylation, while methionine oxidation was designated as a variable modification. All datasets were filtered to a 1% false discovery rate (FDR) at the levels of peptide spectrum match (PSM)/Precursor, Peptide, and Protein.

A proteomic experiment for site‐specific reader identification was performed as described in the *General procedure for proteomic experiment*. 2.5 mM peptide **5** was added to Group A. An Extra 200 µM H3K4me3 or H3K9me3 peptide was added to Group B. Proteomic experiment for inhibitor evaluation was performed as described in the *General procedure for proteomic experiment*. 2.5 mM peptide **12** was added to Group A. An Extra 200 µM MS31 (MedChemExpress, HY‐125837A, 40 mM in DMSO) was added to Group B. Proteomic experiment for reader profiling in the proteome was performed as described in the *General procedure for proteomic experiment*. 2.5 mM peptide **12** was added to Group A. An Extra 7.5 mM peptide 13 was added to Group B.

### Bioinformatic Study

Methyllysine reader domain‐containing proteins in the human genome were identified using the InterPro database (https://www.ebi.ac.uk/interpro/).^[^ ^67^ ^]^ The “search by text” function was used to individually query domain names associated with known or putative methyllysine‐binding activity. For each domain, the corresponding InterPro entry was selected, and the taxonomy filter was applied to restrict results to Homo sapiens (taxon ID: 9606). To ensure data consistency and reproducibility, the search was conducted on November 18, 2023. The following InterPro entries were included in the search: PWWP domain: IPR000313; Chromodomain: IPR017984; BAH domain: IPR001025; Spin domain: IPR042567, IPR003671; Tudor‐like domains: IPR047420, IPR047425, IPR047427, IPR047431, IPR047436; SGF29 Tudor‐like domain: IPR010750; ADD domain: IPR025766; Chromo shadow domain: IPR000953; CW domain: IPR011124; MUM1‐like PWWP domain: IPR035504; PHD finger domain: IPR001965; Tudor domain: IPR002999; WD40 repeat domain: IPR001680. For each InterPro entry, all annotated human proteins were retrieved, including associated UniProt accessions, protein names, protein lengths, and domain locations. The resulting datasets were downloaded and curated. Redundant entries and alternative isoforms were removed, retaining only the primary (canonical) isoform for each gene as annotated in UniProt. The final dataset represents a comprehensive list of human proteins containing at least one methyllysine reader domain.

### Gene Ontology Analysis

Gene ontology analysis was performed using the Metascape online platform.^[^ ^68^ ^]^ The analysis included three gene sets: 22 unreported candidates, 24 known readers, and 556 predicted readers, with *Homo sapiens* set as the species background. Enrichment of biological process (BP), cellular component (CC), and molecular function (MF) ontologies was carried out. GO terms with a *p*‐value< 0.05, a minimum count of 3 genes, and an enrichment factor >1.5 were considered statistically significant.

### Validation of Inhibitor Proteomic Experiments

For SPIN1 and SPIN2B found in the volcano plot, 5 sets of samples were designed, respectively. The first was a negative control containing only the protein without the probe; the second was a positive control with probe but no competition; the third was a vehicle control with DMSO at the same concentration (0.5%) as in the inhibitor treated group; the fourth was the experimental group with 200 µM MS31 for competition, and the fifth with 200 µM H3K4me3 peptide for competition. All these samples with 2.5 mM general probe, 10 µM protein, and other additives were crosslinked by **the** *General procedure for crosslinking* for 30 min. After crosslinking, 4× loading buffer was added to the samples, which were then heated at 95 °C for 8 min and subjected to sodium dodecyl sulfate polyacrylamide gel electrophoresis (SDS‐PAGE). Subsequently, the proteins were transferred to polyvinylidene fluoride (PVDF) membranes, and Ponceau staining was used for loading control. After washing with 1×TBST buffer, the membranes were blocked with 5% BSA at room temperature for 1 h, followed by incubation with streptavidin‐HRP antibody (Thermo Fisher, 21130) at room temperature for another hour. Finally, the membranes were washed with 1×TBST buffer three times, and the protein bands were detected by enhanced chemiluminescence reagent.

### Statistical Analysis

For the comparative analyses in proteomic experiments, statistical significance was determined using Student's *t*‐test. The analysis was performed on the intensity values from n = 3 biologically independent replicates. A two‐tailed *p* value ≤ 0.05 was considered to indicate statistical significance.

## Conflict of Interest

The authors declare no conflict of interest.

## Author Contributions

M.W. conceived the idea and secured funding. J.Y. synthesized the probes, performed crosslinking assays, and proteomic experiments. Y.X. completed the bioinformatic study and provided ulp1. Y.G. expressed and purified the readers and provided methylated peptides for competition. J.Y. prepared the figures and wrote the manuscript with the guidance of M.W., and all authors contributed to the editing of the manuscript.

## Supporting information

Supporting Information

Supporting Information

Supporting Information

## Data Availability

The data that support the findings of this study are available in the supplementary material of this article.
