# Supplementary material for: Development of Dimethylsulfonium Probes for Broad Profiling of Methyllysine Reader Proteins
Source: Adv Sci (Weinh). 2025 Nov 29;13(9):e17751. doi: 10.1002/advs.202517751 (PMC12904088; doi:10.1002/advs.202517751)
Supplement: Supplementary file 1 — Supporting Information [file ADVS-13-e17751-s001.pdf]

Supporting Information

Development of dimethylsulfonium probes for broad profiling of methyllysine reader proteins

Jinyu Yang[a][b], Yihang Xiao[b], Yingxiao Gao[b], Mingxuan Wu*[b][c][d]

Summary of peptides in the study

No.	Label	Sequence	Mass spectrometry data	
			Calculated	Found
**1**		(PEG)$_3$-GGNle$_C$S$^+$me2GG-NH$_2$	684.3055 [M]$^+$	684.3029
**2**	dbio-(PEG)$_3$-GGNle$_C$S$^+$me2GG	desthiobiotin-(PEG)$_3$-GGNle$_C$S$^+$me2GG-NH$_2$	880.4267 [M]$^+$	880.4271
**3**	FLAG-(PEG)$_3$-GGNle$_C$S$^+$me2GG	DYKDDDDK-(PEG)$_3$-GGNle$_C$S$^+$me2GG-NH$_2$	839.8504 [M+H]$^{2+}$	839.8423
**4**	dbio-KG$_6$Nle$_C$S$^+$me2G$_2$-NH$_2$	desthiobiotin-KGGGGGGNle$_C$S$^+$me2GG-NH$_2$	989.4655 [M]$^+$	989.4568
**4'**	dbio-KG$_6$Met$^+$meG$_2$-NH$_2$	desthiobiotin-KGGGGGGMet$^+$meGG-NH$_2$	943.4778 [M]$^+$	943.6168
**5**	dbio-RKG$_6$Nle$_C$S$^+$me2G$_2$-NH$_2$	desthiobiotin-RKGGGGGGNle$_C$S$^+$me2GG-NH$_2$	1145.5666 [M]$^+$	1145.5714
**6**	dbio-RKRG$_6$Nle$_C$S$^+$me2G$_2$-NH$_2$	desthiobiotin-RKRGGGGGGNle$_C$S$^+$me2GG-NH$_2$	1301.6678 [M]$^+$	1301.6654
**7**	dbio-RKRKG$_6$Nle$_C$S$^+$me2G$_2$-NH$_2$	desthiobiotin-RKRKGGGGGGNle$_C$S$^+$me2GG-NH$_2$	715.385 [M+H]$^{2+}$	715.4007
**8**	dbio-DEG$_6$Nle$_C$S$^+$me2G$_2$-NH$_2$	desthiobiotin-DEGGGGGGNle$_C$S$^+$me2GG-NH$_2$	1105.4401 [M]$^+$	1105.4038
**9**	dbio-DEDEG$_6$Nle$_C$S$^+$me2G$_2$-NH$_2$	desthiobiotin-DEDEGGGGGGNle$_C$S$^+$me2GG-NH$_2$	1349.5096 [M]$^+$	1349.4763
**10**	dbio-DREKG$_6$Nle$_C$S$^+$me2G$_2$-NH$_2$	desthiobiotin-DREKGGGGGGNle$_C$S$^+$me2GG-NH$_2$	1389.6362 [M]$^+$	1389.6292
**11**	dbio-RKG$_6$Nle$_C$S$^+$me2G$_4$-NH$_2$	desthiobiotin-RKGGGGGGNle$_C$S$^+$me2GGGG-NH$_2$	1259.6096 [M]$^+$	1259.6113
**12**	biotin-RKG$_6$Nle$_C$S$^+$me2G$_2$-NH$_2$	biotin-RKGGGGGGNle$_C$S$^+$me2GG-NH$_2$	1175.5231 [M]$^+$	1175.5222
**13**	RKG$_6$Kme3G$_2$	RKGGGGGGKme3GG	928.5435 [M+H]$^+$	928.626
**S1**		(PEG)$_3$-GGCGG-NH$_2$	596.2708 [M+H]$^+$	596.213
**S2**		desthiobiotin-(PEG)$_3$-GGCGG-NH$_2$	792.392 [M+H]$^+$	792.316
**S3**	FLAG-(PEG)$_3$-GGCGG	DYKDDDDK-(PEG)$_3$-GGCGG-NH$_2$	1590.6588 [M+H]$^+$	1590.523
**S4**	dbio-KG$_6$CG$_2$-NH$_2$	desthiobiotin-KGGGGGGCGG-NH$_2$	901.4309 [M+H]$^+$	901.46
**S4'**	dbio-KG$_6$MG$_2$-NH$_2$	desthiobiotin-KGGGGGGMGG-NH$_2$	929.4622 [M+H]$^+$	929.458
**S5**	dbio-RKG$_6$CG$_2$-NH$_2$	desthiobiotin-RKGGGGGGCGG-NH$_2$	1057.532 [M+H]$^+$	1057.477
**S6**	dbio-RKRG$_6$CG$_2$-NH$_2$	desthiobiotin-RKRGGGGGGCGG-NH$_2$	1213.6331 [M+H]$^+$	1213.656
**S7**	dbio-RKRKG$_6$CG$_2$-NH$_2$	desthiobiotin-RKRKGGGGGGCGG-NH$_2$	1341.728 [M+H]$^+$	1341.725
**S8**	dbio-DEG$_6$CG$_2$-NH$_2$	desthiobiotin-DEGGGGGGCGG-NH$_2$	1017.4054 [M+H]$^+$	1017.412
**S9**	dbio-DEDEG$_6$CG$_2$-NH$_2$	desthiobiotin-DEDEGGGGGGCGG-NH$_2$	1261.475 [M+H]$^+$	1261.508
**S10**	dbio-DREKG$_6$CG$_2$-NH$_2$	desthiobiotin-DREKGGGGGGCGG-NH$_2$	1301.6015 [M+H]$^+$	1301.505
**S11**	dbio-RKG$_6$CG$_4$-NH$_2$	desthiobiotin-RKGGGGGGCGGGG-NH$_2$	1171.5749 [M+H]$^+$	1171.62
**S12**	biotin-RKG$_6$CG$_2$-NH$_2$	biotin-RKGGGGGGCGG-NH$_2$	1087.4884 [M+H]$^+$	1087.434

MS of peptides used in this study

A Sulfonium peptides were characterized by ESI-MS. Fragments were indicated from different C-S bond cleavages in MS.

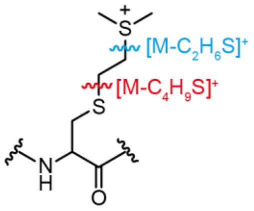

B

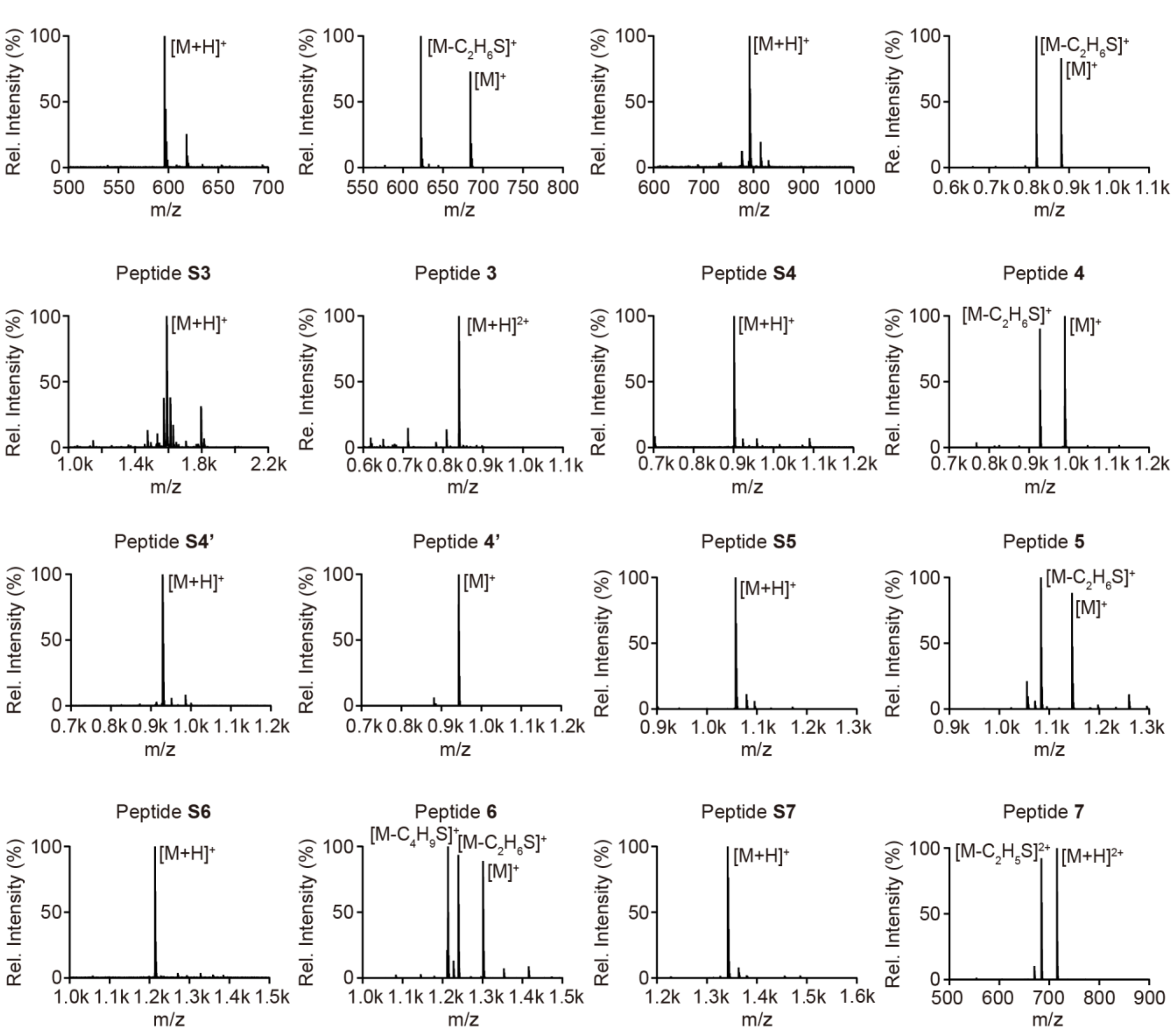

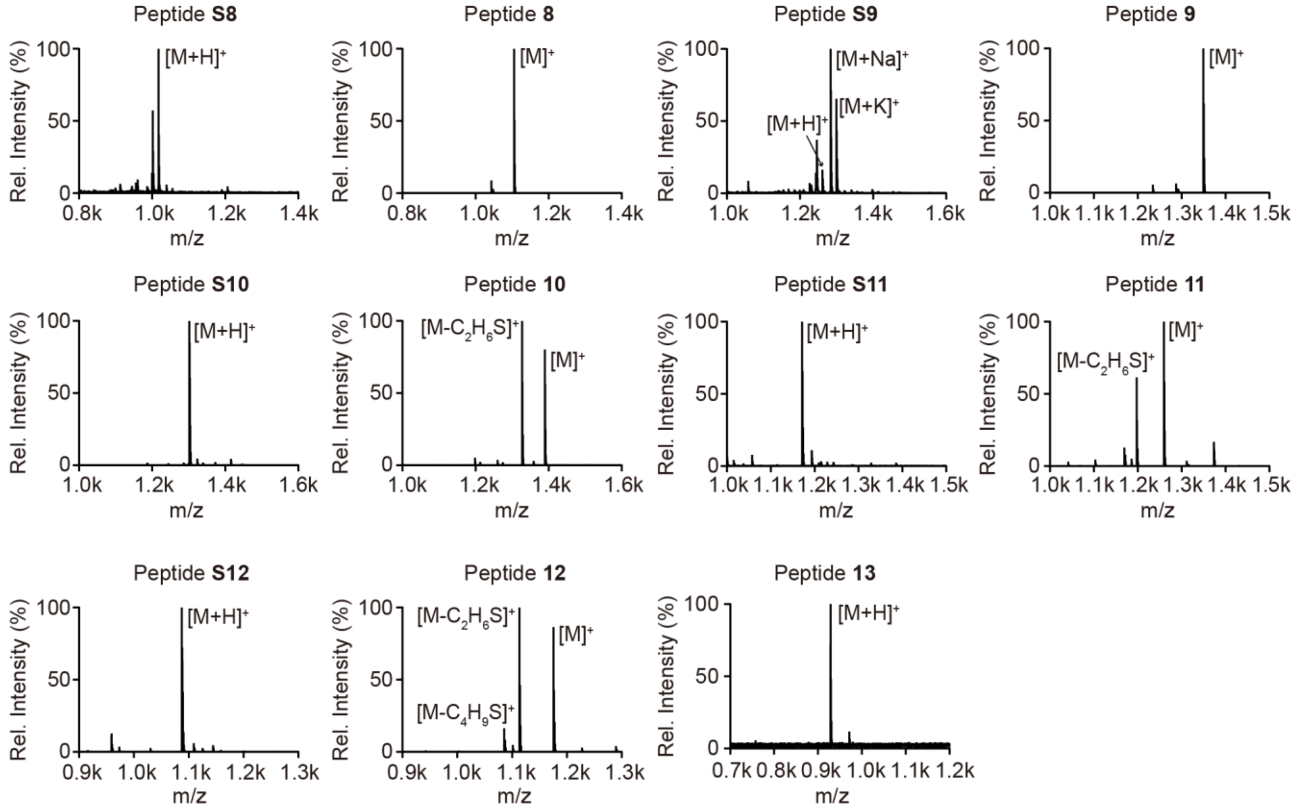
Peptide S8
[M+H]+
Rel. Intensity (%)
100
50
0
0.8k 1.0k 1.2k 1.4k
m/z
Peptide 8
[M]+
0.8k 1.0k 1.2k 1.4k
Peptide S9
[M+Na]+
[M+K]+
[M+H]+
1.0k 1.2k 1.4k 1.6k
Peptide 9
[M]+
1.0k 1.1k 1.2k 1.3k 1.4k 1.5k
Peptide S10
[M+H]+
1.0k 1.2k 1.4k 1.6k
Peptide 10
[M-C2H6S]+
[M]+
1.0k 1.2k 1.4k 1.6k
Peptide S11
[M+H]+
1.0k 1.1k 1.2k 1.3k 1.4k 1.5k
Peptide 11
[M]+
[M-C2H6S]+
1.0k 1.1k 1.2k 1.3k 1.4k 1.5k
Peptide S12
[M+H]+
0.9k 1.0k 1.1k 1.2k 1.3k
Peptide 12
[M-C2H6S]+
[M]+
[M-C4H9S]+
0.9k 1.0k 1.1k 1.2k 1.3k
Peptide 13
[M+H]+
0.7k 0.8k 0.9k 1.0k 1.1k 1.2k

Supporting Figures

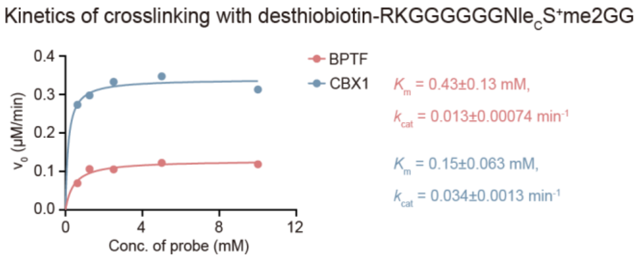

Figure S1. Kinetics of crosslinking with peptide **5**. 10 μM BPTF or CBX1 was treated with peptide **5** at various concentrations for 20 min of crosslinking. The products were quantified by mass spectra, and the K_m and k_{cat} values were calculated through the fitted Michaelis-Menten equation curve.

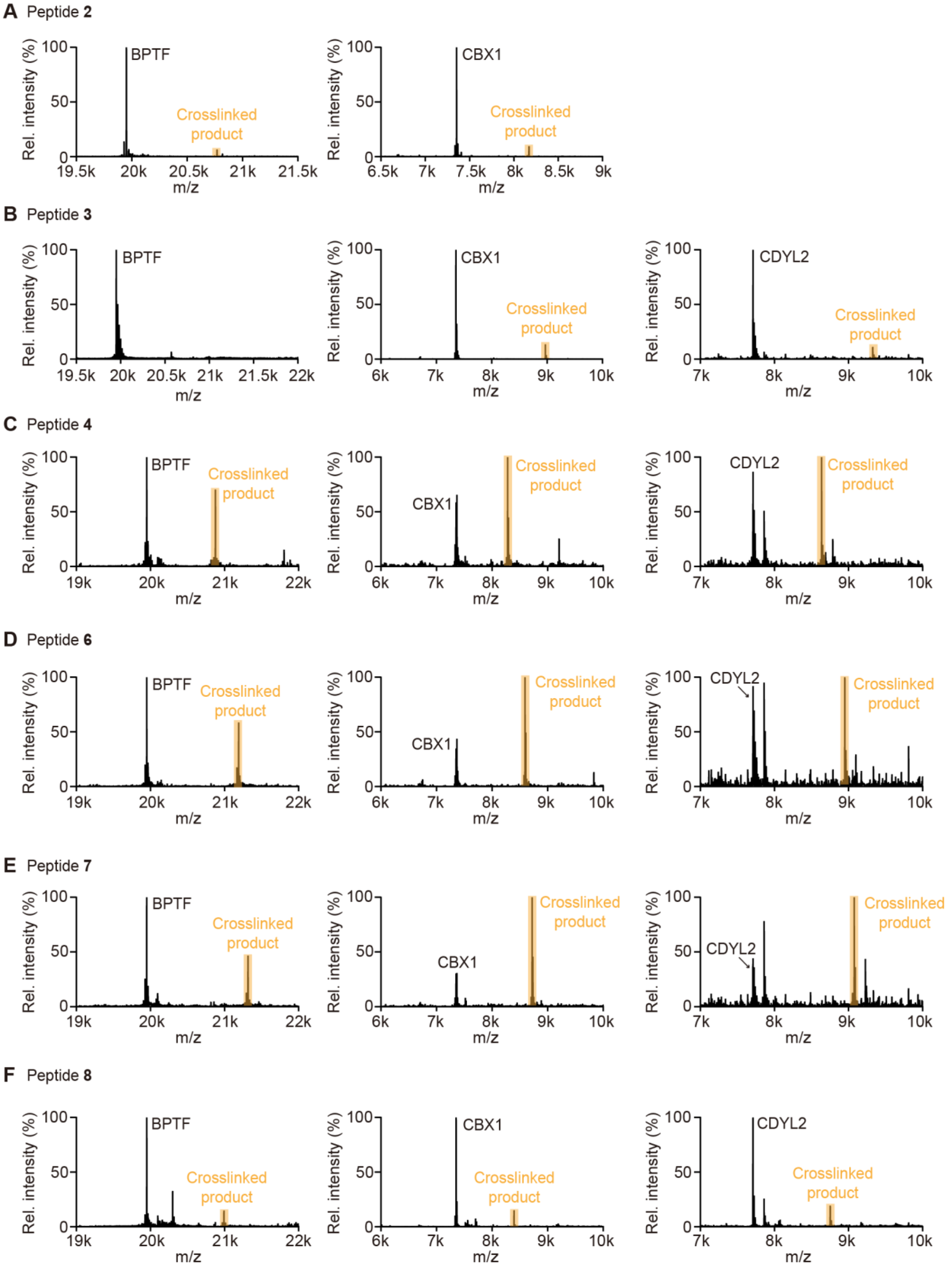
A Peptide 2
B Peptide 3
C Peptide 4
D Peptide 6
E Peptide 7
F Peptide 8
Rel. intensity (%)
m/z
BPTF
CBX1
CDYL2
Crosslinked product
0
50
100
19k
19.5k
20k
20.5k
21k
21.5k
22k
6k
6.5k
7k
7.5k
8k
8.5k
9k
10k

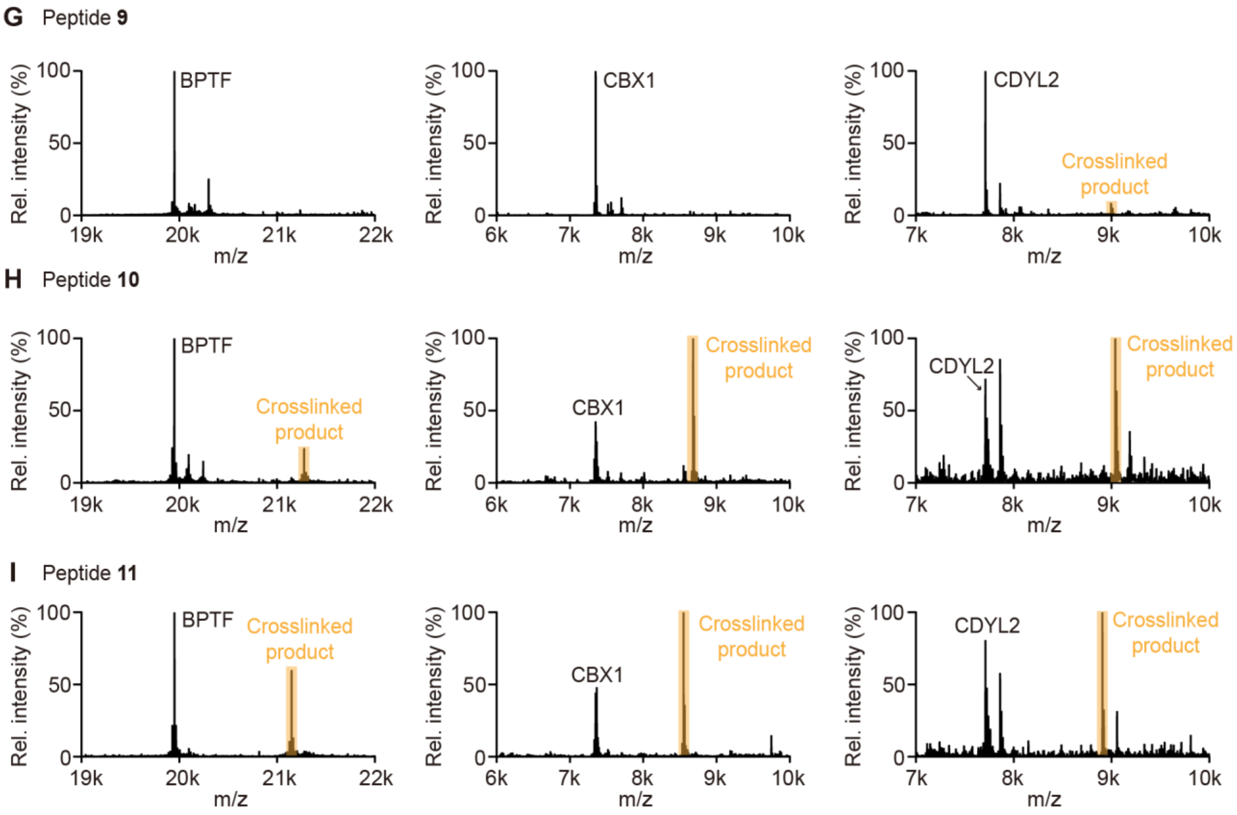

Figure S2. Mass spectra for calculating analytical yields (in Figure 3B) of crosslinking BPTF, CBX1, and CDYL2 with different peptides.

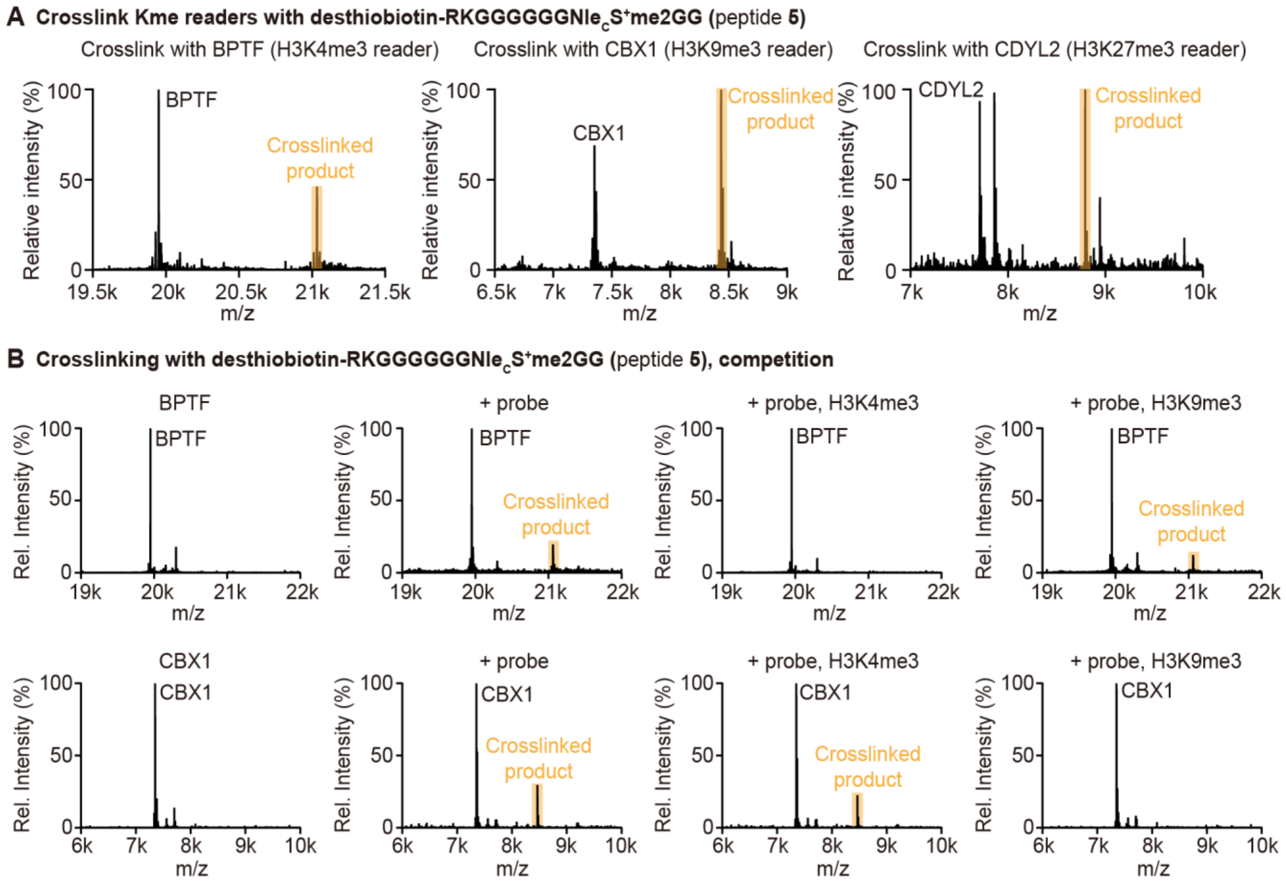

Figure S3. Crosslinking readers with peptide **5**. (A) Crosslinking of peptide **5** with different readers and corresponding high-resolution mass spectra of the reaction mixtures. (B) Crosslinking of peptide **5** with BPTF and CBX1, competition by site-specific methylated peptides, and high-resolution mass spectra of the reaction mixtures.

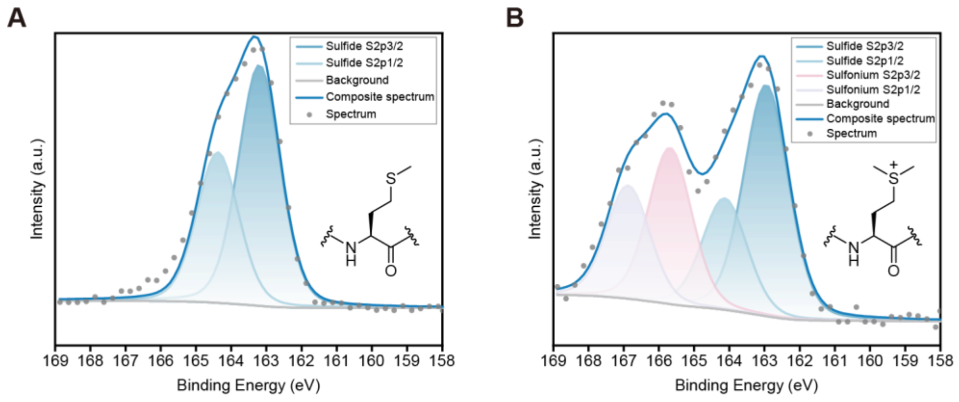

Figure S4. XPS spectrum of peptide **S4’** (A) and peptide **4’** (B).

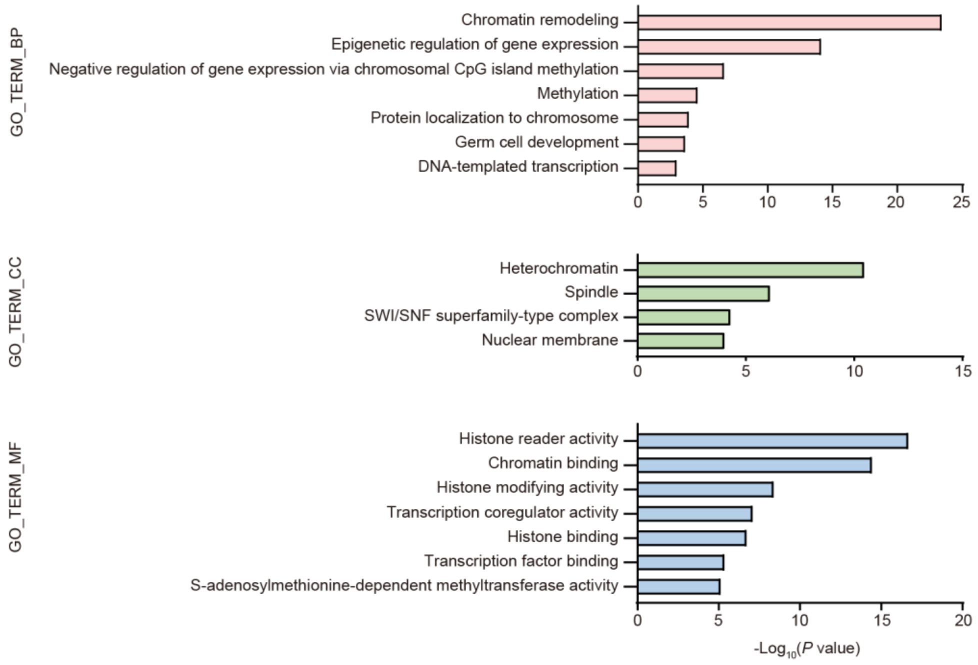

Figure S5. GO analysis of 24 known readers.

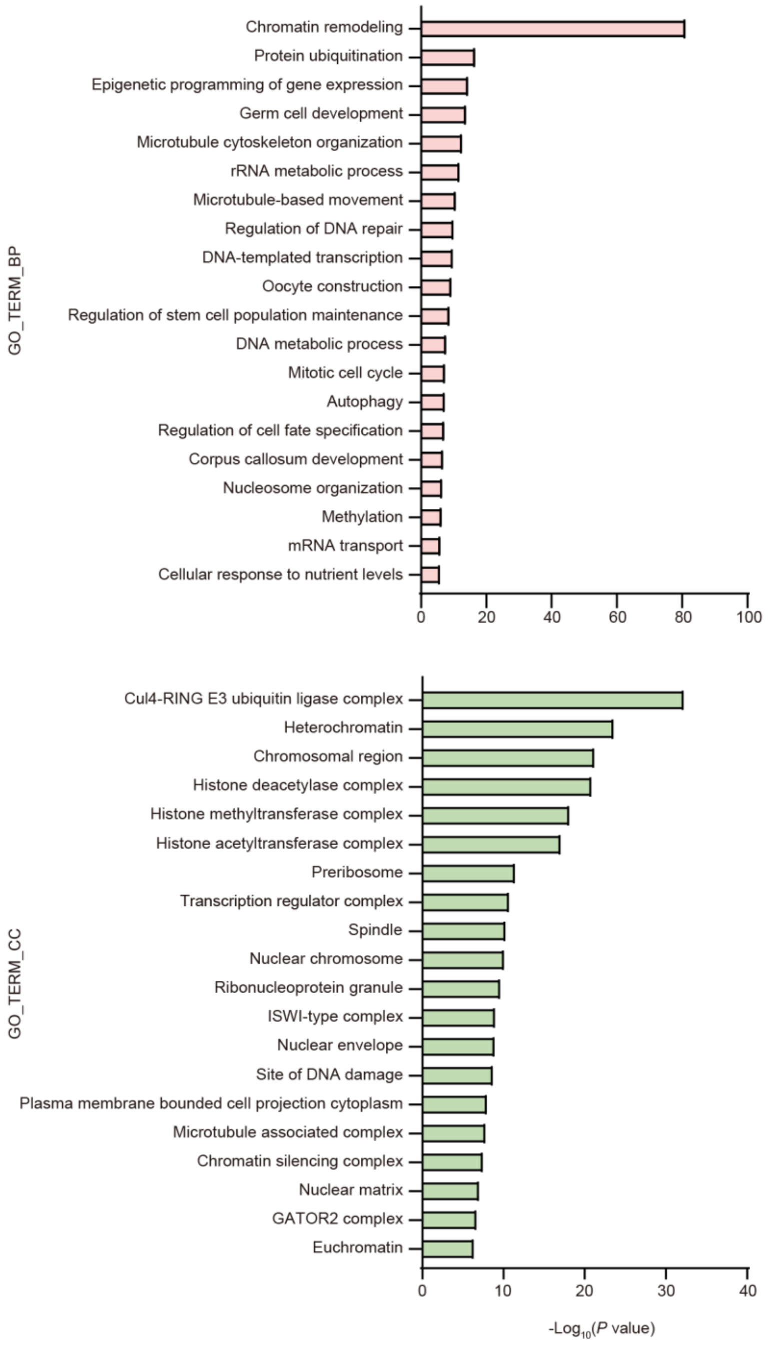
GO_TERM_BP
Chromatin remodeling
Protein ubiquitination
Epigenetic programming of gene expression
Germ cell development
Microtubule cytoskeleton organization
rRNA metabolic process
Microtubule-based movement
Regulation of DNA repair
DNA-templated transcription
Oocyte construction
Regulation of stem cell population maintenance
DNA metabolic process
Mitotic cell cycle
Autophagy
Regulation of cell fate specification
Corpus callosum development
Nucleosome organization
Methylation
mRNA transport
Cellular response to nutrient levels
0
20
40
60
80
100
GO_TERM_CC
Cul4-RING E3 ubiquitin ligase complex
Heterochromatin
Chromosomal region
Histone deacetylase complex
Histone methyltransferase complex
Histone acetyltransferase complex
Preribosome
Transcription regulator complex
Spindle
Nuclear chromosome
Ribonucleoprotein granule
ISWI-type complex
Nuclear envelope
Site of DNA damage
Plasma membrane bounded cell projection cytoplasm
Microtubule associated complex
Chromatin silencing complex
Nuclear matrix
GATOR2 complex
Euchromatin
0
10
20
30
40
-Log10(P value)

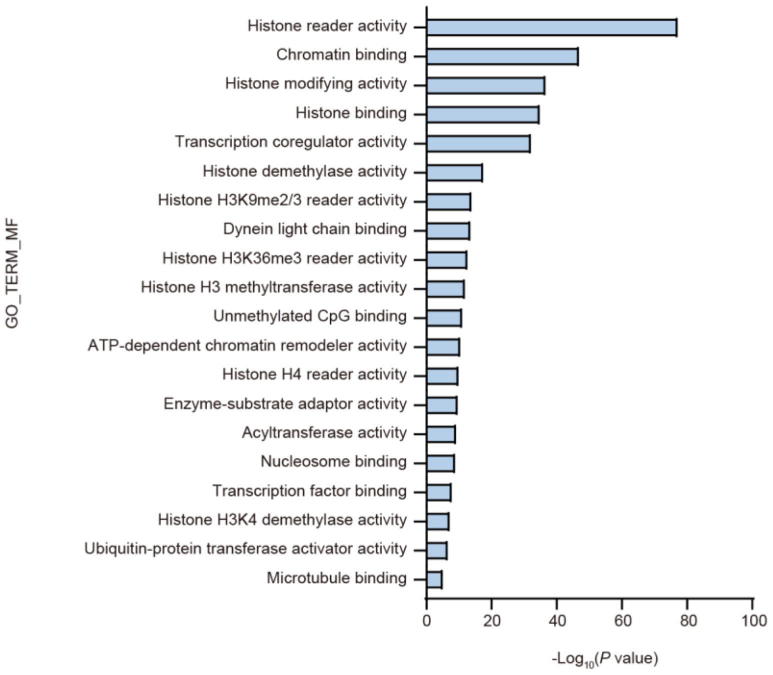

Figure S6. GO analysis of 556 predicted readers.

SUPPORTING INFORMATION

Table S1. Information of 46 hits enriched in the proteomic experiment competed by RKGGGGGGKme3GG.

Accession	Gene symbol	Reader domain	Methylation site
A0A8V8TR54	**CHD3**	Chromo	H3K9
Q9BTC0	**DIDO1**	PHD	H3K4
Q12788	TBL3	WD40	
J3QQK4	**BPTF**	PHD	H3K4
Q7KZF4	SND1	Tudor	
O96028	**NSD2**	PWWP	H3K36
P63244	RACK1	WD40	
A0A8V8TNP6	FXR1	Tudor-like	
Q8WUA4	GTF3C2	WD40	
A0A994J7P9	FXR2	Tudor-like	
Q96T88	**UHRF1**	PHD	H3K9
P29375	**KDM5A**	PHD	H3K4
B3KTM8	**MORF4L1**	Chromo	H3K36
E7ESY4	MTA1	BAH	
Q4LE39	ARID4B	Tudor	
Q9Y6K1	**DNMT3A**	PWWP	H3K36
Q06787	FMR1	Tudor-like	
Q13185	**CBX3**	Chromo	H3K9
O75475	**PSIP1**	PWWP	H3K36
Q9BZ95	**NSD3**	PWWP	H3K36
Q96J01	THOC3	WD40	
H0Y3N9	**PHF8**	PHD	H3K4
Q96QT6	PHF12	PHD	
Q8TD26	CHD6	Chromo	
Q96PU4	**UHRF2**	PHD	H3K9
Q9Y232	**CDYL**	Chromo	H3K9/H3K27
Q5VWG9	**TAF3**	PHD	H3K4
Q8IWS0	PHF6	PHD	
O75530	**EED**	WD40	H3K27
Q969R5	**L3MBTL2**	MBT	H4K20
Q9Y657	**SPIN1**	SPIN	H3K4/H3K9
Q13112	CHAF1B	WD40	
Q9H7E2	TDRD3	Tudor	
J3KS05	**CBX1**	Chromo	H3K9
A0A7I2V5F1	DNMT1	BAH	
H0Y6H0	KDM1B	CW	
A0A087X169	**PHF19**	Tudor	H3K36
Q96ES7	**SGF29**	Tudor-like	H3K4
Q9H7Z6	KAT8	Chromo	
P29374	ARID4A	Tudor	
Q9BPZ2	**SPIN2B**	SPIN	H3K4
A0A8Q3SIG2	**DNMT3B**	PWWP	H3K36
Q92993	**KAT5**	Chromo	p53K372
O75529	TAF5L	WD40	
A0A3B3IT59	MSL3	Chromo	
O14686	KMT2D	PHD	

Green bold font indicates reported reader proteins.
